# Comparative Genomics Reveals Conserved Bacteriocin-Associated Loci in *Lacticaseibacillus paracasei* Strains with Anti-MRSA Activity Isolated from Palmyra Palm Sugar

**DOI:** 10.3390/antibiotics15101000

**Published:** 2026-10-09

**Authors:** Chonticha Romyasamit, Komwit Surachat, Nawanwat C. Pattaranggoon, Morteza Saki, Phoomjai Sornsenee

**Affiliations:** 1 Department of Medical Technology, School of Allied Health Sciences, Walailak University, Thasala District, Nakhon Si Thammarat 80160, Thailand; chonticha.ro@wu.ac.th (C.R.); nawanwat.pa@wu.ac.th (N.C.P.); 2 Research Center for Microbiome, Systems Biology, and Medical Innovation, Walailak University, Thasala District, Nakhon Si Thammarat 80160, Thailand; 3 Research Center in Tropical Pathobiology, Walailak University, Thasala District, Nakhon Si Thammarat 80160, Thailand; 4 Department of Biomedical Sciences and Biomedical Engineering, Faculty of Medicine, Prince of Songkla University, Hat Yai 90110, Thailand; komwit.s@psu.ac.th; 5 Department of Microbiology, Faculty of Medicine, Ahvaz Jundishapur University of Medical Sciences, Ahvaz, Iran; mortezasaki1981@gmail.com; 6 Department of Family and Preventive Medicine, Faculty of Medicine, Prince of Songkla University, Hat Yai 80160, Thailand

**Keywords:** *Lacticaseibacillus paracasei*, bacteriocins, comparative genomics, pangenome analysis, anti-MRSA activity, biofilm inhibition

## Abstract

**Background:** Palmyra palm sugar harbors lactic acid bacteria (LAB) with antimicrobial and probiotic properties. *Lacticaseibacillus paracasei* strains WU0904 and WU2302 inhibit methicillin-resistant *Staphylococcus aureus* (MRSA) and reduce biofilm formation. In this study, we aimed to characterize the distribution and architecture of bacteriocin-associated loci across *L. paracasei*. **Methods:** A screening set of 67 genomes was filtered at ≥95% average nucleotide identity (ANI) and dereplicated at ≥99.9% ANI, yielding 44 nonredundant *L. paracasei* genomes. **Results:** Pangenome analysis identified 7213 gene clusters, comprising 1848 core, 230 soft core, 1528 shell, and 3607 cloud clusters. AntiSMASH analysis identified two major bacteriocin-associated regions in each WU strain. The larger region contained a conserved multipeptide bacteriocin-associated locus comprising predicted prebacteriocins, class II bacteriocins, and genes associated with immunity, regulatory, processing, and export, whereas the smaller region contained a two-gene Blp-family locus. The nine-component backbone of the large locus was complete in 34/44 genomes (77.3%), whereas the small Blp-family locus was present in 18/44 genomes (40.9%). The 59-aa accessory candidate, group_2723, was restricted to WU2302, DmW_181, and CBA7104 and was identical in sequence and syntenic position across these genomes. Phylogenetic analyses showed significant clustering of the small Blp-family locus and group_2723, whereas the large-locus backbone was more broadly distributed. Structural prediction indicated predominantly α-helical conformations among the prioritized bacteriocin candidates. **Conclusions:** The study findings reveal a shared bacteriocin-associated biosynthetic architecture comprising a conserved backbone and lineage-restricted components, providing specific candidates for future functional validation.

## 1. Introduction

Methicillin-resistant *Staphylococcus aureus* (MRSA) remains a major cause of difficult-to-treat infections in healthcare, community, veterinary, and food settings. *S. aureus* causes a broad spectrum of diseases, ranging from superficial skin and soft-tissue infections to pneumonia, osteomyelitis, infective endocarditis, bloodstream infections, and device-associated infections [1,2]. Methicillin resistance is primarily mediated by acquisition of the *mecA* gene or, less frequently, the *mecC* gene, both of which encode alternative penicillin-binding proteins with reduced affinity for most β-lactam antibiotics. PBP2a retains transpeptidase activity and enables peptidoglycan crosslinking even when native penicillin-binding proteins are inhibited [3]. The significance of MRSA to public health is further underscored by its contribution to the global burden of antimicrobial resistance. A systematic analysis estimated that bacterial antimicrobial resistance was directly responsible for approximately 1.27 million deaths and was associated with 4.95 million deaths worldwide in 2019, with methicillin-resistant *S. aureus* among the leading pathogen–drug resistance combinations [4,5].

MRSA infections are commonly treated with vancomycin or daptomycin, whereas linezolid, ceftaroline, and other agents may be selected based on the site and severity of the infection, the antimicrobial susceptibility profile, and patient-specific factors [6]. However, resistance or reduced susceptibility has emerged to each of these therapeutic options. Intermediate vancomycin resistance is commonly associated with cell wall thickening and altered cell envelope homeostasis, whereas high-level vancomycin resistance may result from acquisition of the *vanA* operon [7,8]. Reduced susceptibility to daptomycin has been linked to alterations in membrane charge, phospholipid distribution, and cell envelope stress responses. Linezolid resistance may arise through mutations in 23S rRNA or ribosomal protein genes, as well as through acquisition of transferable resistance determinants such as *cfr* and *optrA*. Mutations affecting PBP2a may also reduce susceptibility to ceftaroline. These resistance mechanisms, together with treatment-related toxicity, limited drug penetration at certain infection sites, and the emergence of persistent bacteremia, constrain the long-term effectiveness of conventional antibiotic therapies [7,8]. MRSA biofilm formation begins with bacterial adhesion to a surface, followed by bacterial aggregation, matrix production, and maturation into a structured community. This biofilm lifestyle promotes bacterial persistence and reduces susceptibility to antimicrobial agents and host defenses [9].

The therapeutic challenge is further exacerbated by the ability of MRSA to form biofilms on tissues, chronic wounds, indwelling catheters, prosthetic materials, and food-contact surfaces. Biofilm-associated cells are embedded in an extracellular matrix and exhibit altered metabolic activity, stress responses, heterogeneous growth states, and increased persistence. These properties can reduce penetration and susceptibility to antibiotics, protect bacteria from host immune responses, and promote chronic or recurrent infections [10]. Thus, successful management may necessitate prolonged antimicrobial therapy, the removal of infected devices, surgical debridement, or combinations of antimicrobial agents. These limitations have increased the need to discover complementary or alternative strategies, including bacteriophages, antimicrobial peptides, anti-virulence compounds, biofilm-disrupting agents, probiotics, and probiotic products.

Lactic acid bacteria (LAB) are promising sources of alternative antimicrobial agents because of their long history of safe use in fermented foods, broad distribution in food- and host-associated microbiota, and ability to produce a variety of bioactive metabolites. These metabolites include organic acids, hydrogen peroxide, fatty acids, biosurfactants, and ribosomally synthesized antimicrobial peptides known as bacteriocins [11]. Bacteriocins have attracted considerable attention owing to their potent antimicrobial activities against foodborne and clinically important pathogens, including multidrug-resistant bacteria. Bacteriocins are diverse in terms of their amino acid sequences, molecular sizes, post-translational modifications, target specificities, and modes of action [11]. Many exert their antimicrobial activity by damaging bacterial membrane integrity through pore formation or membrane depolarization, whereas others target specific membrane receptors, interfere with peptidoglycan biosynthesis, or inhibit essential intracellular processes. Because bacteriocins are genetically encoded, their biosynthetic pathways can be identified through genome mining and further characterized using comparative genomics, heterologous expression, and peptide engineering. These properties have generated growing interest in their application as natural food preservatives, microbiome modulators, antibiotic adjuvants, and alternatives to conventional antimicrobials [12].

Members of the genus *Lacticaseibacillus*, established during the reclassification of the former broadly defined *Lactobacillus* genus, are commonly found in fermented foods, plant-associated habitats, and the gastrointestinal tract [13]. Strains of this genus have been studied for their tolerance to gastrointestinal stress, epithelial adhesion, carbohydrate utilization, pathogen inhibition, and production of antimicrobial metabolites. Palm-derived fermentations represent ecologically relevant reservoirs because their high carbohydrate concentrations and dynamic physicochemical conditions select for microorganisms adapted to osmotic, acidic, and fermentation-associated stresses. Genomic characterization of *L. paracasei* strains isolated from fermented palm sap revealed genes associated with carbohydrate metabolism, stress tolerance, probiotic functions, and antimicrobial potential [14]. However, large catalogs of probiotic-associated genes do not necessarily identify the determinants of a given antimicrobial phenotype. Targeted genomic analysis of strains with experimentally demonstrated antimicrobial activity is warranted to explore potential genotype–phenotype associations.

Our previous study characterized lactic acid bacteria (LAB) isolated from traditional Palmyra palm sugar in southern Thailand. Among 26 isolates obtained from 30 samples, WU0904 and WU2302 were selected based on their probiotic and antimicrobial properties. Both strains survived simulated gastrointestinal conditions and exhibited autoaggregation and adhesion to Caco-2 cells [15]. Notably, both showed strong activity against *S. aureus* and MRSA, with inhibition zones classified as +++ (>16 mm). Culture supernatants from the selected probiotic strains also significantly inhibited MRSA biofilm formation by 82.81–87.24% (*p* < 0.05) [15]. MIC and MBC values were not determined; therefore, a direct quantitative comparison of antimicrobial potency between WU0904 and WU2302 was not possible.

Whole-genome sequencing (WGS) and comparative genome analyses offer rational approaches to answer these questions. Bacteriocin-associated loci often contain multiple functionally coordinated genes, rather than a single precursor peptide sequence. Depending on the class of bacteriocins, such regions may encode one or more precursor peptides, processing enzymes, ATP-binding cassette transporters, accessory secretion proteins, regulatory components, and immunity determinants to protect the producer strain [16,17]. The identification of functional loci remains challenging because bacteriocin precursors are often encoded by short open reading frames that can be missed or misannotated. Moreover, transporters, regulatory proteins, membrane proteases, and immunity-like proteins may have functions other than bacteriocin production. Therefore, similarity to a named bacteriocin or detection of an isolated bacteriocin-associated gene is not sufficient to prove the production of an active antimicrobial peptide. Specialized genome-mining platforms such as antiSMASH [18] have been used to identify ribosomally synthesized and post-translationally modified peptide regions and other secondary metabolite biosynthetic loci.

However, computational predictions are hypothesis-generating and require evaluation of gene organization, conserved genomic context, and peptide sequences. Comparative genomics can increase confidence by examining whether candidate loci are conserved across related isolates and differentiating between broadly conserved components and lineage-specific accessory variations. Pangenome and phylogenomic analyses also allow evaluation of the prevalence and phylogenetic distribution of candidate precursors, immunity, regulation, processing, and export genes. Structural prediction can provide an extra layer of description by testing whether prioritized peptide candidates adopt conformations consistent with known classes of bacteriocins. However, the genomic conservation and predicted peptide structures do not confirm their expression, secretion, maturation, or antimicrobial activity, and all in silico findings require biochemical validation.

This study investigated the genetic basis of the previously demonstrated anti-MRSA activities of *L. paracasei* WU0904 and WU2302 using comparative genomics, integrating whole-genome sequencing, pangenome analysis, phylogenomics, and bacteriocin-focused genome mining. The genomes were taxonomically resolved and compared to a dedicated collection of publicly available *L. paracasei* genomes to define their evolutionary relationships and genomic diversity. Conserved bacteriocin-associated loci were characterized at the gene cluster and peptide levels to identify biosynthetic architectures potentially associated with the antimicrobial phenotype. Representative candidate peptides were subjected to structural prediction to evaluate characteristic features of bacteriocins. Collectively, this study provides genomic insights into conserved bacteriocin-associated loci that may contribute to the reported anti-MRSA phenotype and identifies candidate peptide systems for future biochemical and functional studies.

## 2. Results

### 2.1. Genome Characteristics, Annotation, and Taxonomic Assignment of WU0904 and WU2302

**Figure 1:**
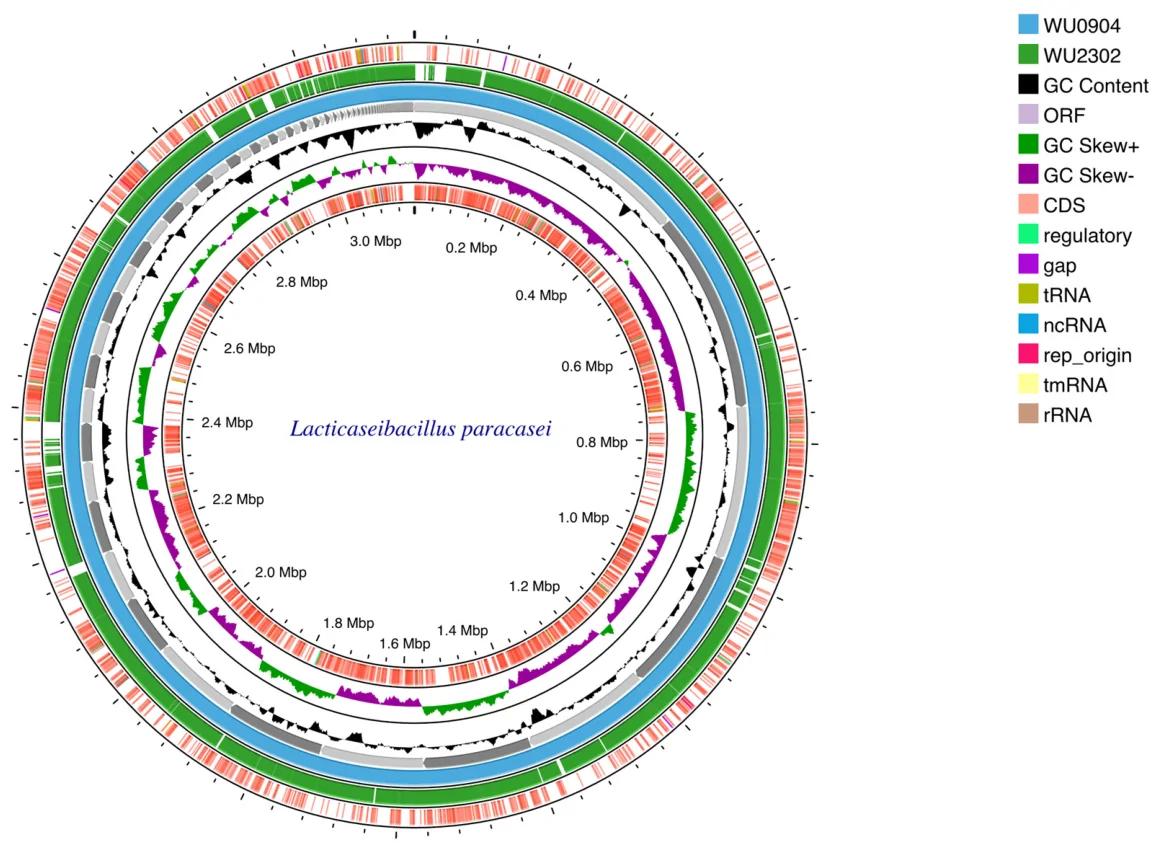
Comparative genome visualization of *Lacticaseibacillus paracasei* WU0904 and WU2302. Concentric tracks represent the draft genome assemblies of WU0904 and WU2302 together with annotated genomic features, GC content, and GC skew. The two genomes display broadly similar genome-wide organization. Genome coordinates are shown in megabase pairs (Mbp). Proksee was used for visualization.

The draft genome sizes of WU0904 and WU2302 were 3.15 Mb and 3.04 Mb, respectively, with similar GC contents of 46.11–46.27% (Table 1). WU0904 comprised 215 contigs with an N50 of 158,377 bp and a largest contig of 437,549 bp, whereas WU2302 comprised 130 contigs with an N50 of 126,405 bp and a largest contig of 330,839 bp. Bakta annotation predicted 2998 protein-coding sequences in WU0904 and 2901 in WU2302. Both genomes contained 56 tRNA genes and one tmRNA, whereas four and five rRNA genes were identified in WU0904 and WU2302, respectively. Comparative genome visualization revealed broadly similar genome-wide organizations and GC profiles between the two strains (Figure 1).

Genome-based taxonomic assignments using GTDB-Tk identified both isolates as *L. paracasei*. The closest reference genome was GCF_000829035.1, with ANI values of 98.41% and 98.21% for WU0904 and WU2302, respectively, and corresponding alignment fractions of 0.837 and 0.839. Both assemblies met the predefined quality thresholds and were retained for subsequent comparative genomic analyses (Table 1).

### 2.2. Comparative Genome Collection, Species Filtering, Dereplication, and Pangenome Structure

An initial NCBI for Biotechnology Information search retrieved 981 *L. paracasei* genome records. After metadata deduplication and preliminary quality filtering, 493 nonredundant records were retained, of which 468 met the initial eligibility criteria. Niche-balanced selection of 60 public genomes, together with four type-material outgroups and two focal strains, yielded a preliminary collection of 66 genomes. Following the addition of the GTDB-matched reference genome GCF_000829035.1, species-level ANI filtering retained 63 *L. paracasei* genomes and excluded four non-*paracasei* outgroups. Subsequent dereplication at ≥99.9% ANI removed 19 near-duplicate genomes, resulting in a final comparative dataset of 44 nonredundant *L. paracasei* genomes, including WU0904 and WU2302 (Supplementary Tables S1 and S2a–c).

Pangenome analysis of the 44 genomes identified 7213 gene clusters (Table 2; Supplementary Table S3). Among these, 1848 (25.6%) were classified as core, 230 (3.2%) as soft core, 1528 (21.2%) as shell, and 3607 (50.0%) as cloud gene clusters. Together, core and soft-core clusters accounted for 28.8% of the pangenomes, whereas 71.2% of the clusters belonged to the shell or cloud fractions.

### 2.3. Shared and Strain-Associated Gene Content

WU0904 and WU2302 shared 2496 Panaroo gene clusters, whereas 380 clusters were detected only in WU0904 and 360 only in WU2302. Among the shared gene content, two clusters annotated as a GNAT-family acetyltransferase and an alpha/beta hydrolase were present in both focal strains but absent in all 42 public *L. paracasei* genomes in the comparative dataset. Examination of their genomic neighborhoods revealed variable regions containing mobile- or phage-associated features. None of the clusters overlapped with the bacteriocin-associated regions identified by antiSMASH, and no direct association with the previously reported antimicrobial phenotype was evident from the present genomic analysis. A quantitative summary of the shared and strain-associated gene content is provided in Supplementary Table S3.

### 2.4. Bacteriocin-Associated Regions and Conserved Locus Architecture

**Figure 2:**
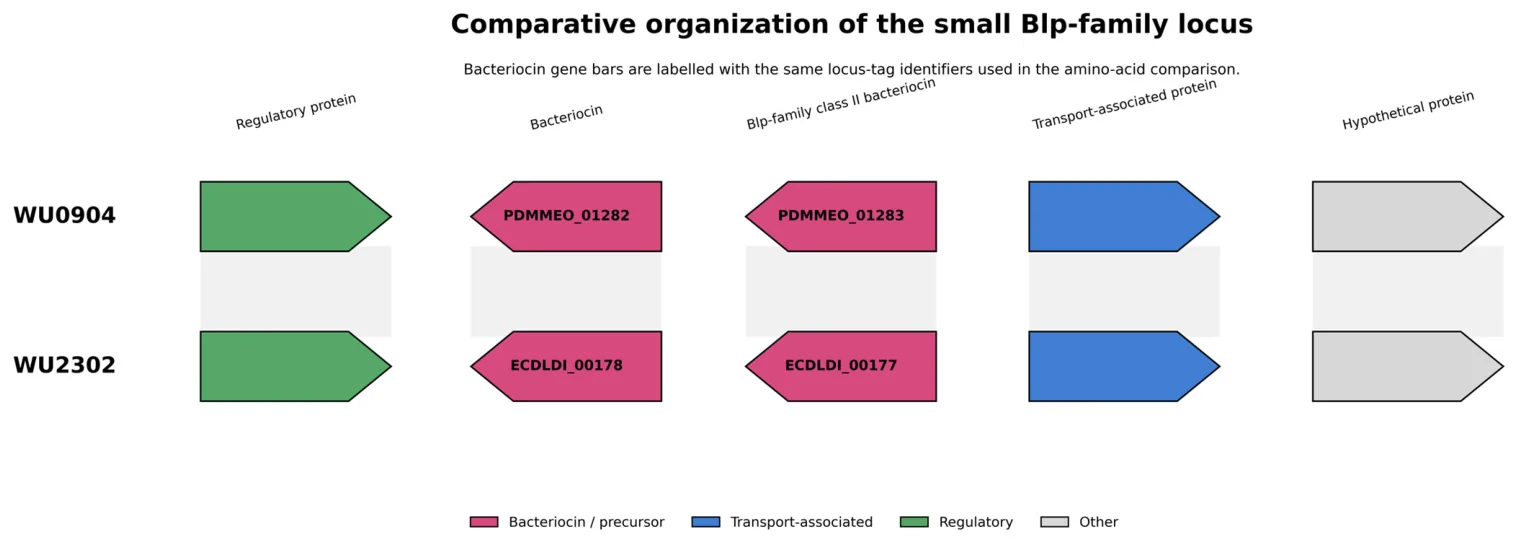
Comparative organization of the small Blp-family bacteriocin-associated locus in *Lacticaseibacillus paracasei* WU0904 and WU2302. Corresponding regions indicate conserved organization of the bacteriocin and Blp-family class II bacteriocin genes, with high nucleotide similarity across the aligned locus.

AntiSMASH analysis identified two major bacteriocin-associated regions in each focal strain. The smaller region contained adjacent genes annotated as bacteriocins and Blp-family class II bacteriocins. A comparison of the corresponding regions in WU0904 and WU2302 revealed approximately 10.3 kb of aligned sequence with a weighted nucleotide identity of 95.45%, indicating substantial conservation of the small Blp family locus between the two strains (Table 3; Figure 2).

**Figure 3:**
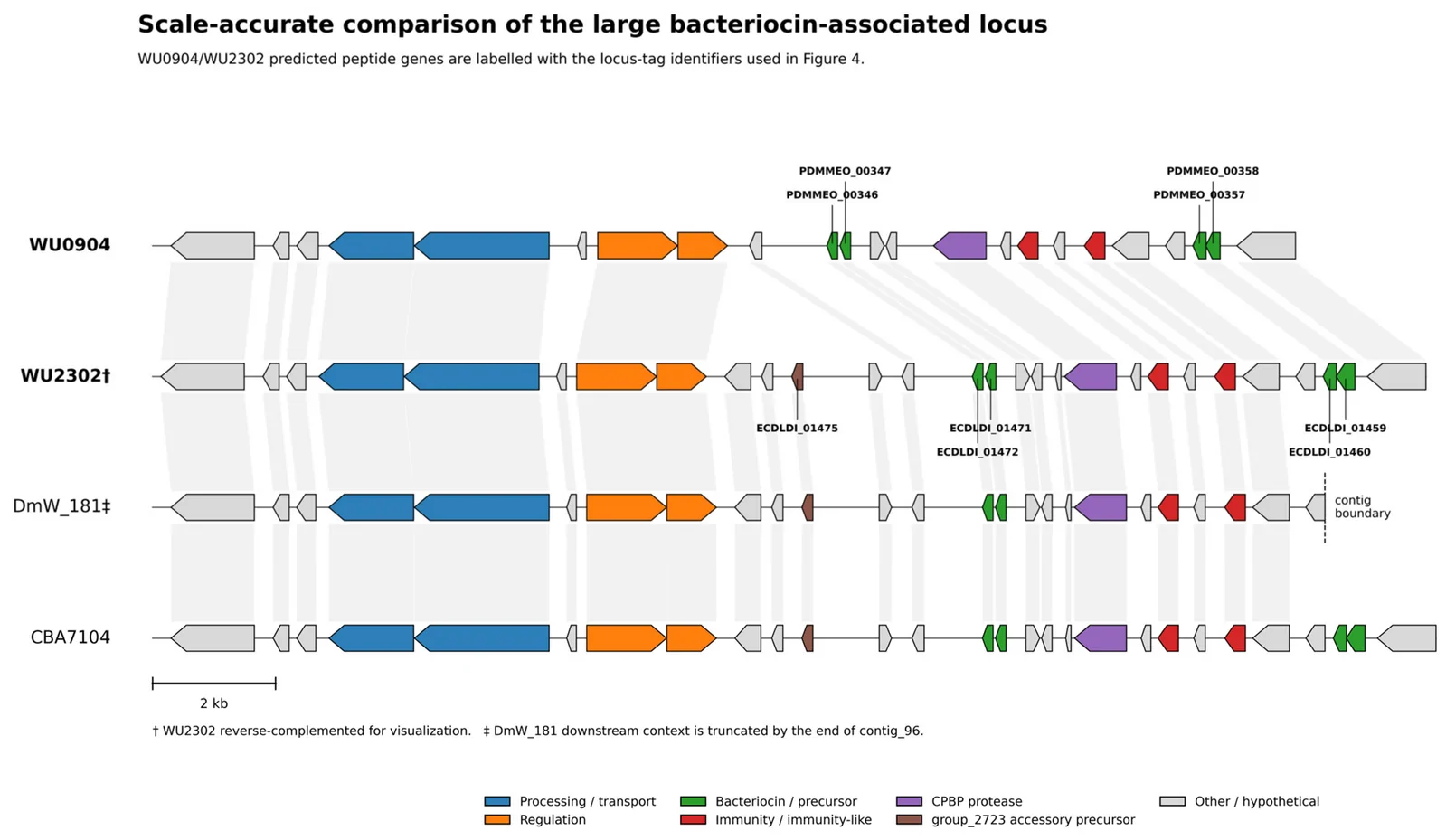
Comparative organization of the large bacteriocin-associated locus in *Lacticaseibacillus paracasei* WU0904 and WU2302. Homologous genes and functional modules are aligned between the two strains, illustrating conservation of the major bacteriocin-associated backbone together with variation in accessory components. WU2302 contains an additional predicted 59-aa bacteriocin candidate that is absent from the corresponding WU0904 locus. The gene map is schematic and is not drawn to scale.

The larger region comprised a multipeptide bacteriocin-associated locus containing multiple predicted prebacteriocins, class II bacteriocins with double-glycine leaders, a ComC/BlpC-family peptide, a bacteriocin immunity protein, a Lactococcin A ABC transporter family-like permease, and a LagD processing/export ATPase. Approximately 19.4 kb of this region aligned between WU0904 and WU2302, with a weighted nucleotide identity of 97.6%. Despite high sequence identity across the aligned portions, variations in accessory sequence content were observed, including an additional predicted 59-aa bacteriocin candidate in WU2302 (Table 3; Figure 3). Notably, the putative immunity gene in WU0904 and WU2302 was oriented opposite to the adjacent CAAX-family protease gene, suggesting local organizational variation compared with other strains bearing the large bacteriocin-associated locus. No significant matches to known biosynthetic gene clusters were identified in these regions using antiSMASH. Detailed regional comparisons are presented in Supplementary Table S4.

### 2.5. Peptide-Level Conservation and Precursor Variation

Pairwise comparisons of homologous bacteriocin-associated proteins between WU0904 and WU2302 showed high amino acid sequence conservation (95.5–100%) (Table 4; Supplementary Table S5). PDMMEO_00357 and ECDLDI_01460 were identical across 71 aa, whereas PDMMEO_00358 and ECDLDI_01459 shared 98.7% identity across the 77-aa aligned region. ECDLDI_01459 contained an additional 72 bp upstream coding segment, consistent with a putative 24-aa N-terminal extension; however, the translation start site requires experimental validation.

**Figure 4:**
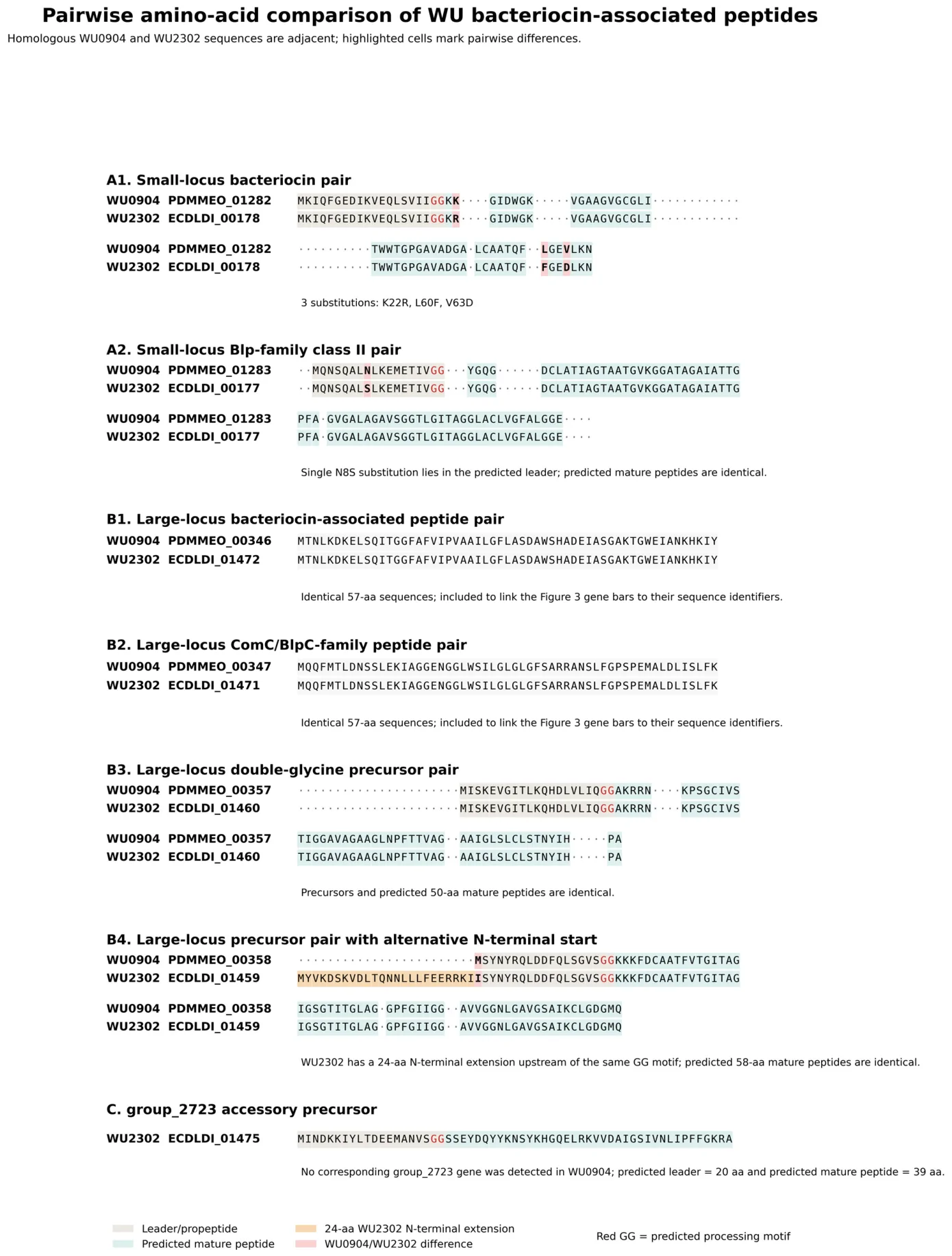
Pairwise amino-acid comparison of predicted WU bacteriocin-associated peptides. (**A1**) Small-locus bacteriocin pair; (**A2**) small-locus Blp-family class II pair. (**B1**) Large-locus bacteriocin-associated peptide pair; (**B2**) large-locus ComC/BlpC-family peptide pair; (**B3**) large-locus double-glycine precursor pair; (**B4**) large-locus precursor pair with an alternative N-terminal start. (**C**) group_2723 accessory precursor ECDLDI_01475. Homologous WU0904 and WU2302 sequences are shown adjacent, with pairwise differences highlighted. Leader/propeptide regions and predicted mature-peptide regions are indicated as shown in the figure. The 24-aa N-terminal extension of WU2302 ECDLDI_01459 is highlighted, and its predicted 58-aa mature peptide is identical to that of WU0904 PDMMEO_00358. Processing sites of the WU candidates are sequence-based predictions and have not been experimentally verified.

Comparison with reviewed bacteriocins with annotated mature peptides showed that the predicted WU mature peptides were generally divergent from known bacteriocins, although the mature peptide of PDMMEO_01283/ECDLDI_00177 showed greater similarity to the ThmA component of thermophilin 13 (Table 4; Figure 4). For PDMMEO_00358 and ECDLDI_01459, the predicted cleavage model yielded leader regions of 19 and 43 aa, respectively, but an identical 58-aa predicted mature peptide. The unusually long predicted leader of ECDLDI_01459 requires experimental confirmation.

The rare accessory candidate ECDLDI_01475 (group_2723) contained a predicted 20-aa double-glycine leader followed by a 39-aa mature peptide. Although its processing architecture resembled those of reviewed double-glycine bacteriocin precursors, its predicted mature peptide showed a maximum global identity of only 27.9% to the reviewed reference set, supporting its classification as a divergent predicted class II-like accessory bacteriocin precursor.

### 2.6. Distribution of Bacteriocin-Associated Loci Across the 44-Genome Dataset

The nine-component backbone of the large bacteriocin-associated locus was complete in 34 of the 44 genomes (77.3%), including WU0904 and WU2302. The two-gene small Blp family locus showed a more restricted distribution and was detected in 18 genomes (40.9%), including both focal strains. In contrast, the 59-aa accessory bacteriocin candidate represented by group_2723 was detected in only three genomes (6.8%), WU2302, DmW_181, and CBA7104, and was absent from WU0904. The genome-level presence/absence calls for bacteriocin-associated traits are shown in Supplementary Table S6a.

### 2.7. Rare Accessory Bacteriocin Candidate Within a Conserved Locus Variant

The group_2723 proteins ECDLDI_01475 (WU2302), LKFAEK_02746 (DmW_181), and GJIBJJ_02465 (CBA7104) were identical across all 59 amino acids. In all three genomes, the corresponding gene occupied the same syntenic position within the large bacteriocin-associated locus adjacent to the ComC/BlpC-family peptide genes, regulatory components, LagD-associated processing/export machinery, and Lactococcin A ABC transporter permease. Complete sequence conservation and shared syntenic positions of group_2723 across the three genomes identified a distinct accessory configuration of the large bacteriocin-associated locus.

### 2.8. Phylogenetic Distribution of Bacteriocin-Associated Traits

**Figure 5:**
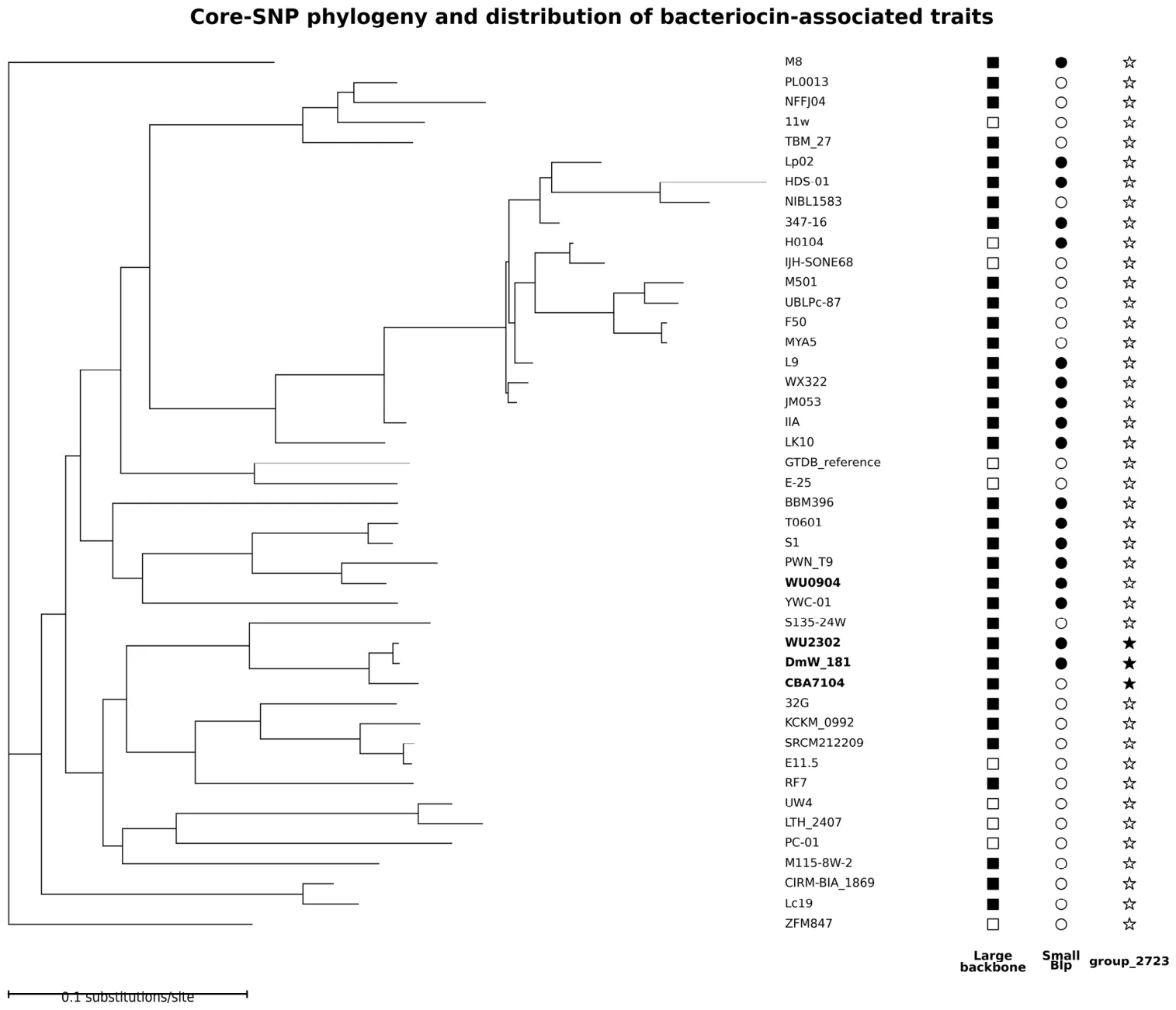
Core-SNP phylogeny of the 44 Lacticaseibacillus paracasei genomes annotated with bacteriocin-associated traits. Filled symbols indicate the presence of the complete nine-component large-locus backbone (“Large backbone”), the two-gene small Blp-family locus (“Small Blp”), and the group_2723 accessory precursor candidate. The small Blp-family locus and group_2723 showed significant phylogenetic clustering by both mean patristic-distance and Fitch-parsimony analyses, whereas the large-locus backbone showed significant lineage-associated structure only by Fitch-parsimony analysis.

The complete large-locus backbone was broadly distributed across the phylogeny (Figure 5). Trait-positive genomes did not show a significantly smaller mean pairwise patristic distance than that expected under random permutations (observed: 0.2817; permutation mean: 0.2862; *p* = 0.1511). In contrast, Fitch parsimony analysis indicated fewer trait-state changes than expected under random distribution (observed, 6; permutation mean, 9.17; *p* = 0.0059), indicating some lineage-associated structures in the presence or absence of the large-locus backbone.

The small Blp family locus showed significant phylogenetic clustering based on both measures. The observed mean patristic distance among positive genomes was 0.2500, compared with a permutation mean of 0.2862 (*p* = 0.0024), whereas the observed number of Fitch changes was seven, compared with a permutation mean of 14.19 (*p* = 0.0002). The group_2723 accessory bacteriocin candidate showed the strongest phylogenetic clustering, with a mean patristic distance of 0.0236 compared with a permutation mean of 0.2865 (*p* = 0.0002), and only one observed Fitch change compared with a permutation mean of 2.96 (*p* = 0.0004). The full permutation and parsimony statistics are provided in Supplementary Table S6b.

### 2.9. Structural Characterization of Predicted Bacteriocin Peptides

**Figure 6:**
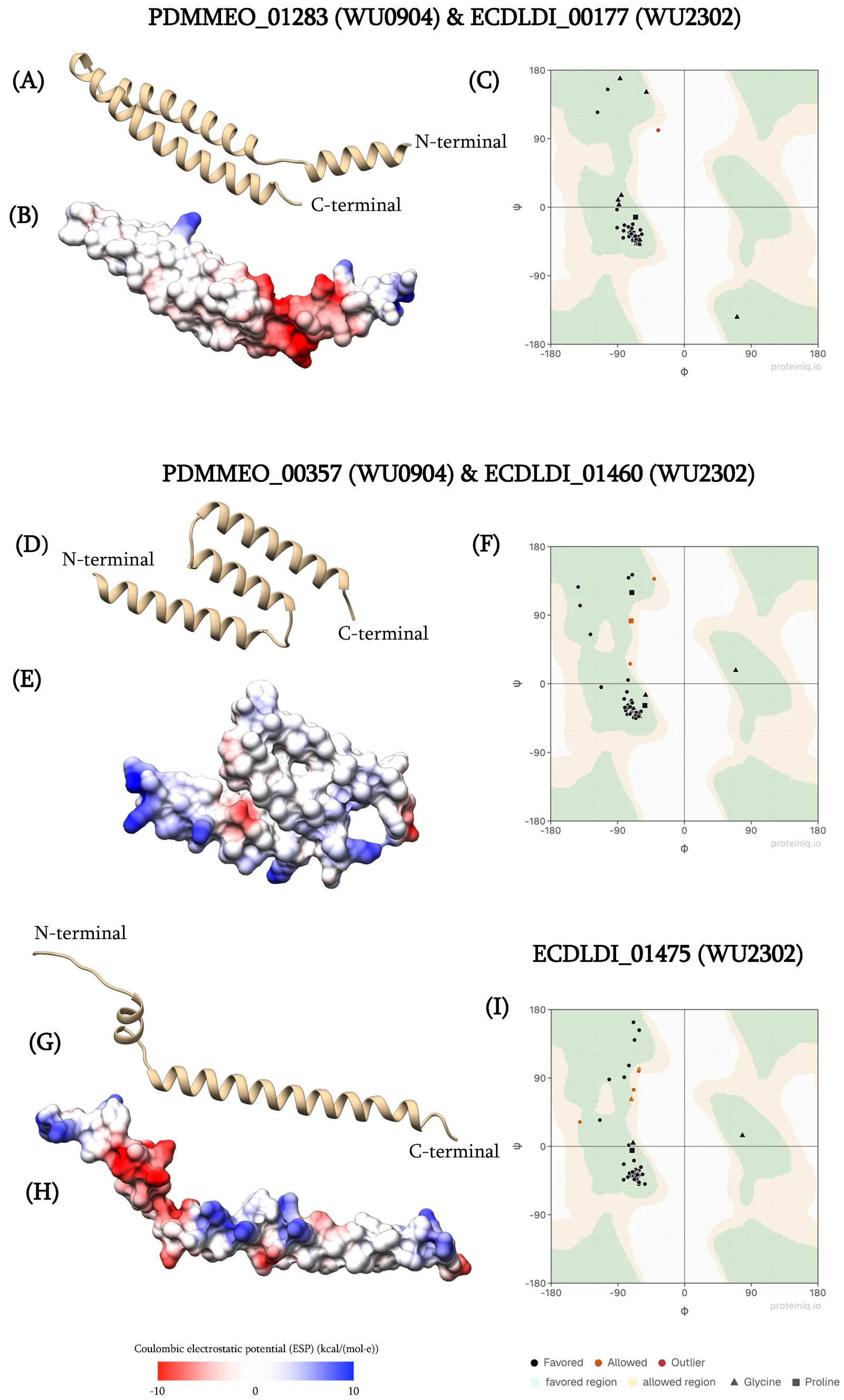
Predicted structures, surface electrostatic potential, and stereochemical validation of representative bacteriocin-associated peptide sequences from WU0904 and WU2302. Ribbon representation, Coulombic electrostatic potential (ESP) surface, and Ramachandran plot are shown for each representative sequence. (**A**–**C**) PDMMEO_01283 (WU0904) and ECDLDI_00177 (WU2302): (**A**) predicted ribbon structure; (**B**) ESP surface; (**C**) Ramachandran plot. (**D**–**F**) PDMMEO_00357 (WU0904), representing the identical PDMMEO_00357/ECDLDI_01460 homologous pair: (**D**) predicted ribbon structure; (**E**) ESP surface; (**F**) Ramachandran plot. (**G**–**I**) ECDLDI_01475 (WU2302): (**G**) predicted ribbon structure; (**H**) ESP surface; (**I**) Ramachandran plot. Three-dimensional structures were predicted using AlphaFold3. Electrostatic potential surfaces were calculated using the ESP module in UCSF Chimera and colored on a continuous scale from red (−10 kcal/mole, negative potential) through white (0 kcal/mole, neutral) to blue (+10 kcal/mole, positive potential). Ramachandran plots were generated using the ProteinIQ web application; residues are classified as favored (black circles), allowed (orange circles), or outliers (red circles), with glycine (triangles) and proline (squares) residues indicated separately.

PDMMEO_01283 (WU0904) and ECDLDI_00177 (WU2302) adopted an extended α-helical fold (Figure 6A) with a predominantly electronegative surface and weak electropositive termini (Figure 6B). Ramachandran analysis showed 98.8% of the residues were in the favored regions, with one outlier (Tyr19) and a pTM score of 0.62 (Figure 6C; Supplementary Figure S2).

PDMMEO_00357 (WU0904) and ECDLDI_01460 (WU2302) formed a compact multihelix bundle (Figure 6D) with a largely neutral-to-electropositive surface and a localized negative patch (Figure 6E). Ramachandran analysis showed 95.7% favored and 4.3% allowed residues, with no outliers; the pTM score was 0.34 (Figure 6F; Supplementary Figure S2).

ECDLDI_01475 (WU2302) formed an elongated helical structure (Figure 6G) with a pronounced electronegative potential and a small electropositive terminal patch (Figure 6H). Ramachandran analysis showed 91.1% favored and 8.9% allowed residues, with no outliers; the pTM score was 0.41 (Figure 6I; Supplementary Figure S2).

Overall, the models showed distinct electrostatic profiles with predominantly electronegative elongated structures and a more electropositive compact bundle. The relatively low pTM scores of the two models indicate uncertainty in their global folds, supporting further refinement by energy minimization and molecular dynamics simulations before interpreting their potential roles in the molecular mechanisms of antibacterial activity.

## 3. Discussion

The previously demonstrated anti-MRSA activities of WU0904 and WU2302 provide a phenotypic basis for interpreting the genomic findings of this study. Both strains inhibited MRSA, and their culture supernatants markedly reduced MRSA biofilm formation. Comparative genomic analysis revealed two bacteriocin-associated loci shared by both strains, comprising a highly conserved large multipeptide locus and a smaller Blp-family locus, together with a 59-aa group_2723 accessory candidate detected only in WU2302. Although group_2723 was detected in WU2302 but not WU0904, its presence cannot currently be linked to broader or stronger antimicrobial activity. Both strains showed similarly strong activity against *S. aureus* and MRSA in our previous study, and no direct quantitative comparison between the strains was performed. Therefore, the contribution of group_2723 to the antimicrobial spectrum or potency of WU2302 requires functional validation. The large bacteriocin-associated locus was present in 34 of 44 genomes (77.3%), suggesting that it is a relatively conserved genomic feature rather than a strain-specific determinant of the anti-MRSA phenotype. Thus, its presence alone is insufficient to explain the antimicrobial activity previously observed in WU0904 and WU2302. Whether this locus contributes to anti-MRSA activity may depend on its expression, peptide processing and secretion, and the activity of the encoded products, all of which require experimental validation. Previous genome-based analysis of *L. paracasei* SD1 similarly identified multiple putative bacteriocin-associated genes, supporting the genomic diversity of bacteriocin-related determinants within this species [19]. The observed anti-MRSA activity could therefore be due to a single bacteriocin, a combination of bacteriocins, or the combined effects of bacteriocins and other non-proteinaceous antimicrobial metabolites in the culture supernatant.

The contribution of bacteriocin-like molecules to the anti-MRSA phenotype is biologically plausible, as bacteriocins produced by LAB have exhibited substantial activity against MRSA and are increasingly recognized as potential alternatives or adjuncts to conventional antimicrobials [20]. Among *L. paracasei* strains, F9-02 produced a bacteriocin with bactericidal activity against MRSA (MIC, 7.56 μg/mL); this activity was abolished by proteolytic treatment but retained after pH neutralization and catalase treatment, supporting a proteinaceous rather than acid- or hydrogen peroxide-mediated effect [21]. Similarly, the bacteriocin LSX01 from *L. paracasei* LS-6 inhibited *S. aureus* (MIC, 16.90 μg/mL) and showed antibiofilm activity [22]. Collectively, these studies demonstrate that LAB can inhibit MRSA through diverse secreted antimicrobial components. These findings are consistent with the anti-staphylococcal and antibiofilm phenotypes previously observed for WU0904 and WU2302. However, unlike these previously characterized single bacteriocins, the WU strains harbored multiple predicted precursor peptides together with immune, regulatory, processing, and export components, suggesting a potentially more complex bacteriocin-associated system.

The organization of the large bacteriocin-associated locus also suggests its potential role in peptide production and secretion. The locus contains several predicted prebacteriocins and class II bacteriocins with double-glycine leaders, as well as a ComC/BlpC-family peptide, an immunity protein, LagD-associated processing/export machinery, and a Lactococcin A ABC transporter permease. This architecture is consistent with that of known class II bacteriocin systems, in which precursors bearing double-glycine leaders are recognized and cleaved by dedicated ABC transporters during export to release mature peptides [23,24]. High conservation of the biosynthetic architecture does not necessarily imply equivalent antimicrobial activity. One predicted class II bacteriocin pair (PDMMEO_00357/ECDLDI_01460) was identical over 71 amino acids, whereas another shared 98.7% identity but contained a 24-aa N-terminal extension in WU2302, supported by an additional 72 bp coding sequence. Such leader variations may affect precursor recognition, processing, or export [23,24], although their functional significance remains unknown. Moreover, the presence of a bacteriocin locus does not establish peptide expression, maturation, or secretion [24,25]. The complete large-locus backbone occurred in 34 of the 44 genomes and was therefore not specific to the two anti-MRSA strains. Thus, the conserved backbone may have biosynthetic potential, whereas variations in the precursor sequences, regulation, processing, or expression may contribute to strain-specific antimicrobial activity.

Membrane disruption is a plausible mechanism if the predicted peptides contribute to the anti-MRSA activities of WU0904 and WU2302. Class II bacteriocins often increase membrane permeability and disrupt ionic homeostasis, whereas two-peptide class IIb bacteriocins can act synergistically to induce ion leakage and impair cellular energetics [24,26]. Similar effects have been demonstrated in *S. aureus*. Plantaricin YKX increases membrane permeability, causes leakage of K+ and intracellular content, disrupts membrane integrity, and inhibits biofilm formation [27]. Similarly, plantaricin GZ1-27 increased membrane permeability and disrupted membrane integrity in MRSA, resulting in cellular damage and death [28].

The multipeptide organization of the large locus is particularly relevant because class IIb bacteriocins often require two complementary peptides for optimal activity. These peptides are typically encoded by adjacent genes, synthesized by double-glycine leaders, and processed and exported by dedicated machinery [26]. Well-characterized systems, including lactococcin G and plantaricins E/F and J/K, demonstrate cooperative activity, in which complementary peptides interact in membrane environments to form functional antimicrobial complexes [24,26,29]. Although the loci in WU0904 and WU2302 cannot yet be classified as functional two-peptide systems, the presence of multiple precursors together with processing, transport, immunity, and regulatory components supports a potential multi-peptide model. Thus, differences in precursor sequences, accessory components, expression, processing, and peptide abundance may contribute to strain-dependent antimicrobial activity despite the conservation of the large-locus backbone.

The small Blp family locus represents a more restricted level of variation than the widespread large backbone. It was present in 18 of the 44 genomes (40.9%), including WU0904 and WU2302, and showed significant phylogenetic clustering based on both the mean patristic distance and Fitch parsimony. The corresponding Blp family class II bacteriocins in WU0904 and WU2302 shared 98.8% amino-acid identity. Blp-family class II bacteriocins have also been annotated in other *L. paracasei* genomes, and experimentally expressed class II bacteriocins from *L. paracasei* CNCM I-5369 showed antimicrobial activity both individually and in combination [30,31]. In *Streptococcus*, Blp-family systems are regulated through quorum sensing, whereby secreted BlpC activates the BlpH–BlpR two-component system and induces bacteriocin and immune gene expression, illustrating how Blp-associated antimicrobial activity can be tightly coupled to regulatory signaling [32]. These findings support the functional potential of the multipeptide bacteriocin repertoire in *L. paracasei*, although the activity of the Blp-family peptides identified here remains to be determined. The lower prevalence and stronger phylogenetic structure of this locus suggest a lineage-associated distribution. However, its relationship with MRSA inhibition cannot be established without phenotypic data for the comparator genomes. The rare group_2723 accessory candidate showed a strong lineage-associated distribution with complete sequence and positional conservation among the three carriers. Importantly, its absence from WU0904 indicates that it cannot explain the anti-MRSA activity shared by WU0904 and WU2302. Instead, ECDLDI_01475 may represent an accessory component that contributes to strain-specific differences in antimicrobial potency or target specificity, making it a priority candidate for functional characterization.

The diverse phylogenetic distributions of bacteriocin-associated components suggest the dynamic evolution of LAB antimicrobial repertoires. Bacteriocin loci can differ substantially, even among closely related strains, through gene gain, loss, recombination, and horizontal transfer, resulting in lineage-specific combinations of conserved and accessory components [33]. Such evolutionary plasticity may promote the diversification of bacteriocin repertoires in response to microbial competition and ecological pressures, while conserved biosynthetic machinery is retained across broader lineages [33,34]. The pattern observed in this study is consistent with this evolutionary model, with a broadly distributed biosynthetic backbone accompanied by more lineage-restricted bacteriocin-associated components. However, phylogenetic clustering alone cannot distinguish between vertical inheritance and differential or horizontal acquisition. Short bacteriocin precursor genes are prone to annotation errors, and fragmented draft assemblies can generate false presence/absence patterns in pangenome analyses [35]. Therefore, the evolutionary history of these loci should be interpreted cautiously until they are supported by complete genome or locus-specific sequence validation.

The structural characterization of the prioritized candidates provides additional insights into their potential functional diversity. The selected peptides represented distinct components of the bacteriocin repertoire, including a Blp family class II bacteriocin (PDMMEO_01283/ECDLDI_00177), a conserved double-glycine leader-containing bacteriocin (PDMMEO_00357/ECDLDI_01460), and a rare accessory candidate, ECDLDI_01475. The predicted structures differed markedly in topology and surface electrostatics: PDMMEO_01283/ECDLDI_00177 and ECDLDI_01475 adopted elongated, predominantly electronegative helical structures, whereas PDMMEO_00357/ECDLDI_01460 formed a compact multihelix bundle with a more electropositive surface. Such differences may be functionally relevant because class II bacteriocins commonly adopt amphipathic α-helical conformations that facilitate membrane association, while complementary peptides in two-peptide systems can interact within the membrane to form active antimicrobial complexes [26,29]. Thus, the contrasting predicted structural and electrostatic properties of the WU candidates provide hypothesis-generating features for future investigation of possible membrane interactions or peptide cooperation; however, these potential mechanisms cannot be established from the structural models alone and require experimental validation. These properties may also be relevant to the previously reported antibiofilm activities of WU0904 and WU2302, as LSX01 from *L. paracasei* LS-6 exhibited both antibacterial and antibiofilm activities [22], whereas plantaricin YKX inhibited *S. aureus* biofilm formation together with membrane disruption [27]. Thus, MRSA biofilm inhibition may have resulted from reduced bacterial viability or peptide-mediated effects on membrane physiology and biofilm development. However, structural predictions alone cannot establish these mechanisms, and peptide-level testing of both planktonic and biofilm-associated MRSA is required.

The main limitation is that genomic prediction alone cannot determine whether the identified peptides mediate the observed anti-MRSA activity. In our previous study, we demonstrated the in vitro anti-MRSA and antibiofilm activities of WU0904 and WU2302 but did not determine whether these activities were mediated by the bacteriocin candidates identified here. Furthermore, the current in silico analyses do not confirm the expression, secretion, maturation, or antimicrobial activity of these peptides. LAB supernatants may contain organic acids, hydrogen peroxide, and other antimicrobial metabolites, emphasizing the need to experimentally distinguish proteinaceous activities. In *L. paracasei* F9-02, antimicrobial activity persisted after pH neutralization and catalase treatment but was abolished by proteolysis, supporting a proteinaceous antimicrobial mechanism [21]. Similar analyses of WU0904 and WU2302, followed by peptide-level characterization, would help determine the contributions of the predicted bacteriocins. The prioritized mature peptides could be tested individually and in combination, and LC-MS/MS of the active supernatant fractions could confirm their natural production. Ultimately, targeted disruption of precursor or processing/export genes would provide stronger causal evidence linking bacteriocin-associated loci to the anti-MRSA phenotype. A further limitation is the fragmented nature of the WU0904 and WU2302 genome assemblies, comprising 215 and 130 contigs, respectively. Assembly fragmentation may affect the detection of short bacteriocin precursor genes and the assessment of locus completeness. Hence, apparent gene absence or differences in locus organization should be interpreted with caution. Future long-read sequencing would help resolve these regions and confirm the complete architecture of the bacteriocin-associated loci.

Overall, our findings complement the previously demonstrated anti-MRSA phenotypes of WU0904 and WU2302 by revealing a shared bacteriocin-associated biosynthetic architecture with additional strain-specific accessory variation. The conserved multipeptide backbone may provide shared biosynthetic potential, whereas the more restricted Blp family locus, precursor variation, and accessory components, such as group_2723, may contribute to additional functional diversity. This interpretation is consistent with the experimentally characterized proteinaceous anti-staphylococcal compounds from other *L. paracasei* strains and established class II bacteriocin mechanisms involving membrane disruption and cooperative peptide activity. However, these genomic associations do not establish a causal relationship with the observed anti-MRSA phenotype. Thus, peptide-level structural and functional validation is essential to determine the components that contribute to the anti-MRSA and antibiofilm activities of WU0904 and WU2302.

## 4. Materials and Methods

### 4.1. Bacterial Strains and Culture Conditions

*L. paracasei* WU0904 and WU2302 strains used in this study were previously isolated from traditional Palmyra palm sugar collected in southern Thailand. Both strains have been previously characterized for probiotic-associated properties, such as tolerance to simulated gastrointestinal conditions, adhesion to Caco-2 intestinal epithelial cells, and antimicrobial and antibiofilm activities against MRSA [15]. For routine propagation, the strains were cultivated in de Man, Rogosa, and Sharpe (MRS) broth (HiMedia, Mumbai, India) at 37 °C for 24 h under anaerobic conditions. Cultures were maintained on MRS agar and stored at −80 °C in MRS broth supplemented with 20% (*v*/*v*) glycerol (Sigma-Aldrich, St. Louis, MO, USA) until use.

### 4.2. DNA Extraction, Whole-Genome Sequencing, Assembly, and Annotation

Genomic DNA from *L. paracasei* WU0904 and WU2302 was extracted using a DNeasy Blood & Tissue Kit (QIAGEN, Hilden, Germany) according to the manufacturer’s instructions. Prior to whole-genome sequencing, DNA concentration and purity were assessed spectrophotometrically based on the A260/A280 ratio and DNA integrity was evaluated by agarose gel electrophoresis.

Purified genomic DNA from both strains was submitted to the Beijing Genomics Institute (BGI, Shenzhen, China) for whole-genome sequencing using the MGISEQ-2000 platform with 150 bp paired-end reads. Raw sequencing data were processed using BacSeq v1.0 pipeline [36], which integrates established bioinformatics tools for bacterial genome assembly, annotation, and quality assessment. De novo genome assembly was performed using SPAdes [37], and assembly quality was evaluated using QUAST [38] based on metrics, including total assembly length, number of contigs, GC content, and N50. Genome completeness was assessed using BUSCO [39]. Initial gene prediction and functional annotation were performed using Prokka v1.2.0 [40] as implemented in the BacSeq workflow.

### 4.3. Public Genome Collection, Taxonomic Verification, Quality Control, and Dereplication

Genome assemblies and associated metadata for *Lacticaseibacillus paracasei* were retrieved from the National Center for Biotechnology Information (NCBI) on 15 July 2026 using the NCBI Datasets command-line interface. The focal-species query was performed using the datasets summary genome taxon with the options --tax-exact-match, --assembly-source all, --assembly-version current, --exclude-atypical, and --mag exclude. The type material genomes of *Lacticaseibacillus casei* and *Lacticaseibacillus rhamnosus* were additionally retrieved using the from-type option and the same assembly filters for use as taxonomic outgroups. Both the RefSeq and GenBank assemblies were included. The initial query yielded 981 *L. paracasei* genomic records.

Genome metadata were parsed using a custom Python 3 workflow, and records were deduplicated based on BioSample accessions where available, paired RefSeq–GenBank accessions, or assembly accessions. Within each duplicate group, representative records were prioritized according to the predefined quality criteria, with preference given to RefSeq (GCF_) over the corresponding GenBank (GCA_) assemblies, followed by assembly level, annotation availability, metadata completeness, and contig number. This procedure yielded 493 nonredundant records, of which 468 met the preliminary eligibility criteria of a genome size of 2.5–3.6 Mb and ≤300 contigs. Ecological niches were assigned by ordered keyword matching across isolation sources, hosts, strains, and related metadata fields. A niche-balanced sampling strategy was subsequently applied to select 60 public genomes representing palm-derived and fermented food, dairy, human or gastrointestinal, animal, environmental, and other eligible sources while maintaining strain-level, geographic, genomic, and metadata diversity.

The selected collection comprised 60 public *L. paracasei* genomes and four type-material outgroup genomes, including two *L. casei* and two *L. rhamnosus* genomes. The addition of WU0904 and WU2302 resulted in a preliminary dataset containing 66 genomes. An additional GTDB-matched reference genome (GCF_000829035.1) was included for species verification, yielding 67 genomes for taxonomic filtering and dereplication. Genomes were retained within the focal *L. paracasei* genomic species when they exhibited an average nucleotide identity (ANI) of ≥95.0% and a minimum directional alignment fraction of ≥50.0% relative to the reference genome. The four *L. casei* and *L. rhamnosus* outgroups did not meet these criteria and were therefore excluded, leaving 63 genomes after species-level filtering.

The remaining genomes were dereplicated at ≥99.9% ANI with a minimum directional alignment fraction of ≥50.0%. Representative selection prioritized the retention of WU0904, WU2302, and the GTDB reference genome, followed by RefSeq status, higher assembly completeness and quality, lower contig counts, and the availability of informative strains and isolation-source metadata. After removing 19 near-duplicate genomes, the final comparative dataset comprised 44 non-redundant *L. paracasei* genomes, including 42 publicly available genomes, along with WU0904 and WU2302. The final assembly quality was assessed using QUAST v5.3.0 with a minimum contig length of 500 bp. Assemblies were required to have genome sizes of 2.5–3.6 Mb, ≤300 contigs, N50 values of ≥10 kb, and GC contents of 45.0–48.0%. Assemblies containing 151–300 contigs or N50 values of 10–30 kb were retained with quality warnings. All 44 genomes satisfied the predefined final quality criteria.

### 4.4. Pangenome and Phylogenomic Analyses 

All 44 dereplicated *L. paracasei* genomes were uniformly re-annotated using Bakta v1.12.0 to minimize annotation-related variations among genomes obtained from different sources. The resulting standardized annotations were used as inputs for pangenome analysis using Panaroo v1.8.0. Gene clusters were classified as core (99–100%; corresponding to presence in all 44 genomes in this dataset), soft core (95–<99%), shell (15–<95%), or cloud (<15%). Gene presence/absence profiles were further used to identify clusters shared between WU0904 and WU2302. Clusters were detected exclusively in either of the two focal strains.

For phylogenomic analysis, a core SNP phylogeny was reconstructed from the 44-genome dereplicated collection. Branch lengths from the resulting Newick tree were retained and used to calculate pairwise patristic distances for subsequent analyses of phylogenetic clustering of bacteriocin-associated traits.

### 4.5. Identification and Comparative Analysis of Bacteriocin-Associated Loci

Biosynthetic gene clusters in WU0904 and WU2302 were predicted using antiSMASH 8.0 [18], with particular emphasis on the bacteriocin-associated regions potentially associated with bacteriocin production.

The predicted regions and surrounding genomic contexts were examined to identify candidate precursor peptides and genes associated with immunity, regulation, peptide processing, and export. The analysis focused on the two principal bacteriocin-associated regions in each focal strain, while the other predicted biosynthetic regions were retained as part of the complete antiSMASH region inventory.

The homologous bacteriocin-associated regions in WU0904 and WU2302 were compared at the nucleotide and gene organization levels. Following the standardization of locus orientation, region-level GenBank files were subjected to nucleotide sequence comparison, and the conservation of gene organization and synteny was visualized using a clinker, a tool for generating gene cluster comparison figures. Sequence alignment, nucleotide identity, conservation of the principal biosynthetic backbone, and variation in accessory components were compared. Candidate precursor peptides and associated proteins were extracted from Bakta and antiSMASH annotations and further compared using amino acid sequence alignment to assess sequence conservation and strain-specific variation.

Therefore, bacteriocin-focused analysis integrates antiSMASH predictions with uniform genome annotation, direct sequence comparison, and conservation of genomic context to characterize the shared and variable features of the candidate loci.

### 4.6. Systematic Comparison and Processing Analysis of Predicted Bacteriocins

Nine focal bacteriocin candidates encoded by the small Blp-family and large bacteriocin-associated loci were compared systematically with reviewed bacteriocin sequences. A curated reference set was obtained from UniProtKB using the query reviewed: true AND protein_name: bacteriocin AND length: [20 TO 150], yielding 70 reviewed bacteriocin records. Precursor processing annotations, including PROPEP, SIGNAL, CHAIN, and PEPTIDE features, were retrieved from UniProt flat files. Thirty-six reviewed proteins contained explicit annotated mature-peptide features and were used for mature-peptide-level comparisons.

Predicted WU precursors were aligned with representative reviewed bacteriocins using MAFFT v7.526 with the L-INS-i strategy (--localpair --maxiterate 1000). Leader regions of the WU candidates were defined provisionally by the N-terminal double-glycine motif, with cleavage predicted immediately after the second glycine. These processing sites were regarded as sequence-based predictions and not as experimentally established cleavage sites. Mature-peptide sequences were extracted independently from the predicted leaders.

Predicted mature peptides were compared with the 36 explicitly annotated reviewed mature bacteriocins using blastp-short and global pairwise alignment. Global alignments were performed with Biopython PairwiseAligner using BLOSUM62, a gap-opening penalty of −10, and a gap-extension penalty of −0.5. Whole-sequence identity was calculated using the number of identical aligned residues divided by the length of the longer peptide.

### 4.7. Distribution and Phylogenetic Clustering of Bacteriocin-Associated Loci

The distribution of bacteriocin-associated loci across the 44-genome *L. paracasei* dataset was assessed by mapping locus tags from WU0904 and WU2302 to the corresponding Panaroo gene clusters. The conserved large-locus backbone was defined by nine shared gene clusters representing the principal peptide, immunity, processing, and export components, whereas the rare accessory peptide represented by group_2723was analyzed separately because it was not part of the conserved backbone. The small Blp-family locus is defined by two adjacent peptide-associated clusters. The presence or absence of these locus components in each genome was subsequently determined using the Panaroo gene presence/absence matrix.

Phylogenetic clustering of bacteriocin-associated traits was evaluated using core-SNP phylogeny. For each trait, pairwise patristic distances among trait-positive genomes were calculated from phylogenetic tree branch lengths. The observed mean patristic distance was compared to a null distribution generated from 10,000 random permutations while preserving the observed number of trait-positive genomes in each permutation. Phylogenetic clustering was independently assessed using Fitch parsimony by comparing the observed minimum number of trait state changes across the tree with the corresponding null distribution generated from 10,000 permutations. The observed mean patristic distances or number of Fitch changes that were lower than expected under the respective permutation distributions were interpreted as evidence of phylogenetic clustering.

### 4.8. Structural Prediction of Candidate Bacteriocin Peptides

Three representative bacteriocin candidates were prioritized for structural modeling to capture distinct genomic contexts identified in the comparative analysis: PDMMEO_01283/ECDLDI_00177 as a conserved homologous pair from the small Blp-family locus, PDMMEO_00357/ECDLDI_01460 as a conserved homologous pair from the large bacteriocin-associated locus, and ECDLDI_01475 (group_2723) as a rare accessory candidate present in WU2302 but absent from WU0904.

**Protein structure prediction.** Three-dimensional structures were predicted for four amino acid sequences: PDMMEO_01283 and PDMMEO_00357 (both from strain WU0904), and ECDLDI_00177 and ECDLDI_01475 (both from strain WU2302). Structural predictions were performed using AlphaFold3 on the AlphaFold Server (https://alphafoldserver.com/, accessed on 1 August 2026).

**Stereochemical validation.** The stereochemical quality of each predicted model was evaluated by generating Ramachandran plots using Protein IQ software v1.0.0 (https://proteiniq.io/app/ramachandran-plot, accessed on 1 August 2026). Residues were classified into favored, allowed, and outlier/disallowed regions of φ/ψ dihedral angle space based on their backbone torsion angles, with glycine and proline residues flagged separately given their distinct conformational preferences.

**Electrostatic surface potential calculation.** The surface electrostatic properties of each predicted structure were characterized by calculating the coulombic electrostatic potential (ESP) using the ESP module in UCSF Chimera. Calculated potentials were mapped onto the molecular surface of each model and displayed on a continuous color scale ranging from −10 kcal/mol·e (red, indicating negative potential) through 0 kcal/mol·e (white, neutral) to +10 kcal/mol·e (blue, indicating positive potential.

## 5. Conclusions

Comparative analysis of 44 dereplicated *Lacticaseibacillus paracasei* genomes revealed a broadly conserved nine-component bacteriocin-associated backbone, a more restricted and phylogenetically clustered two-gene blp family locus, and a rare lineage-associated 59-aa accessory candidate. WU0904 and WU2302 carried both large and small loci, whereas a rare accessory candidate was present only in WU2302. High sequence and synteny conservation between the two strains support a shared bacteriocin-associated biosynthetic architecture with additional precursor and accessory variations that may contribute to functional diversity. These findings provide a genomic framework for interpreting the previously reported anti-MRSA and antibiofilm activities of WU0904 and WU2302 but do not establish causality. Therefore, peptide-level structural and functional validation is required to identify active components and determine their contribution to the observed antimicrobial phenotype. Following functional validation, these bacteriocin-associated candidates may have applications as bioprotective agents for food preservation or as adjunctive antimicrobial strategies against MRSA infections. Further studies are needed to validate their antimicrobial efficacy, safety, and suitability for food and therapeutic applications.

## Figures and Tables

**Table 1:** Genome assembly, annotation, and taxonomic characteristics of WU0904 and WU2302.

Characteristic	WU0904	WU2302
Genome size (bp)	3,153,900	3,039,630
Number of contigs	215	130
Largest contig (bp)	437,549	330,839
N50 (bp)	158,377	126,405
GC content (%)	46.11	46.27
Predicted CDSs	2998	2901
tRNA genes	56	56
rRNA genes	4	5
tmRNA genes	1	1
GTDB-Tk assignment	*Lacticaseibacillus paracasei*	*Lacticaseibacillus paracasei*
Closest GTDB reference	GCF_000829035.1	GCF_000829035.1
ANI to reference (%)	98.41	98.21
Alignment fraction	0.837	0.839

**Table 2:** Pangenome composition of the 44-genome *Lacticaseibacillus paracasei* dataset.

Category	Definition	Gene Clusters, n	Pangenome (%)
Core	99–100%	1848	25.6
Soft core	95–<99%	230	3.2
Shell	15–<95%	1528	21.2
Cloud	<15%	3607	50.0
Total		7213	100.0

**Table 3:** Comparison of bacteriocin-associated regions in WU0904 and WU2302.

Locus	WU0904 Region	WU2302 Region	Approx. Aligned Length	Weighted Nucleotide Identity	Main Feature
Large multi-peptide locus	contig_1.region001	contig_9.region001	19.4 kb	97.6%	Conserved backbone with accessory variation
Small Blp-family locus	contig_5.region001	contig_1.region001	10.3 kb	95.45%	Nearly full-length conserved locus

**Table 4:** Predicted processing architecture and comparison of focal bacteriocin candidates with reviewed bacteriocins.

Candidate(s)	Precursor Length	Predicted Leader	Predicted Mature Peptide	WU Mature-Peptide Relationship	Closest Reviewed Mature Peptide	Global Identity	Interpretation
PDMMEO_01282/ECDLDI_00178	66 aa	20 aa	46 aa	closely related but non-identical	Q48509/reviewed references	≤31.25%	Divergent class II-like bacteriocin pair
PDMMEO_01283/ECDLDI_00177	84 aa	18 aa	66 aa	identical mature peptides	ThmA, thermophilin 13	45%	Conserved WU peptide, divergent from reviewed mature sequences
PDMMEO_00357/ECDLDI_01460	71 aa	21 aa	50 aa	identical	P24022	31.58%	Predicted double-glycine bacteriocin with no close reviewed mature homolog
PDMMEO_00358	77 aa	19 aa	58 aa	identical to ECDLDI_01459 mature peptide	P05834	29.31%	Predicted class II-like precursor
ECDLDI_01459	101 aa	43 aa	58 aa	identical to PDMMEO_00358 mature peptide	P05834	29.31%	24-aa N-terminal leader extension; mature peptide unchanged
ECDLDI_01475/group_2723	59 aa	20 aa	39 aa	WU2302-specific relative to WU0904	P35618, sakacin-P	27.91%	Divergent accessory class II-like precursor; no close reviewed mature homolog

## Data Availability

All data analyzed during this study are included in this published article and its Supplementary Materials.

## Supplementary Materials

The following supporting information can be downloaded at: https://www.mdpi.com/article/10.3390/antibiotics15101000/s1, Supplementary Table S1. Audit of the preliminary 66-genome collection, including accessions, niche classifications, inclusion status, and exclusion reasons. Supplementary Table S2a–c. Assembly quality information for the final dereplicated genome collection; Selected metadata information for the final dereplicated genome collection; taxonomy/ANI information for the final dereplicated genome collection. Supplementary Table S3. Pangenome category counts and focal-isolate shared and strain-associated gene-content summary. Supplementary Table S4. Predicted processing architecture and similarity of focal WU0904/WU2302 bacteriocin candidates to reviewed bacteriocins. Supplementary Table S5. Pairwise amino-acid comparison and revised interpretation of bacteriocin-associated proteins in WU0904 and WU2302. Supplementary Table S6a,b. Genome-level bacteriocin-trait presence/absence matrix; phylogenetic-clustering statistics. Supplementary Table S7. Software, commands, parameters, evidence status, and unresolved reproducibility items. Supplementary Figure S1. Distribution of bacteriocin-associated loci across the 44-genome *Lacticaseibacillus paracasei* dataset. Presence/absence patterns are shown for the nine-component large-locus backbone, the two-gene small Blp-family locus, and the group_2723 accessory bacteriocin candidate across the 44 genomes. Supplementary Figure S2. The predicted TM-score, a confidence metric that AlphaFold outputs alongside each predicted structure.

## Abbreviations

The following abbreviations are used in this manuscript:

ANI	Average nucleotide identity
BGI	Beijing Genomics Institute
MRS	De Man, Rogosa, and Sharpe
ESP	Electrostatic potential
LAB	Lactic acid bacteria
MRSA	Methicillin-resistant *Staphylococcus aureus*
NCBI	National Center for Biotechnology Information
WGS	Whole-genome sequencing
