# Supplementary material for: Comparative Genomics Reveals Conserved Bacteriocin-Associated Loci in Lacticaseibacillus paracasei Strains with Anti-MRSA Activity Isolated from Palmyra Palm Sugar
Source: Antibiotics (Basel). 2026 Oct 9;15(10):1000. doi: 10.3390/antibiotics15101000 (PMC13700875; doi:10.3390/antibiotics15101000)
Supplement: Supplementary file 1 [file antibiotics-15-01000-s001.zip › 20260928 Supplementary figure.pdf]

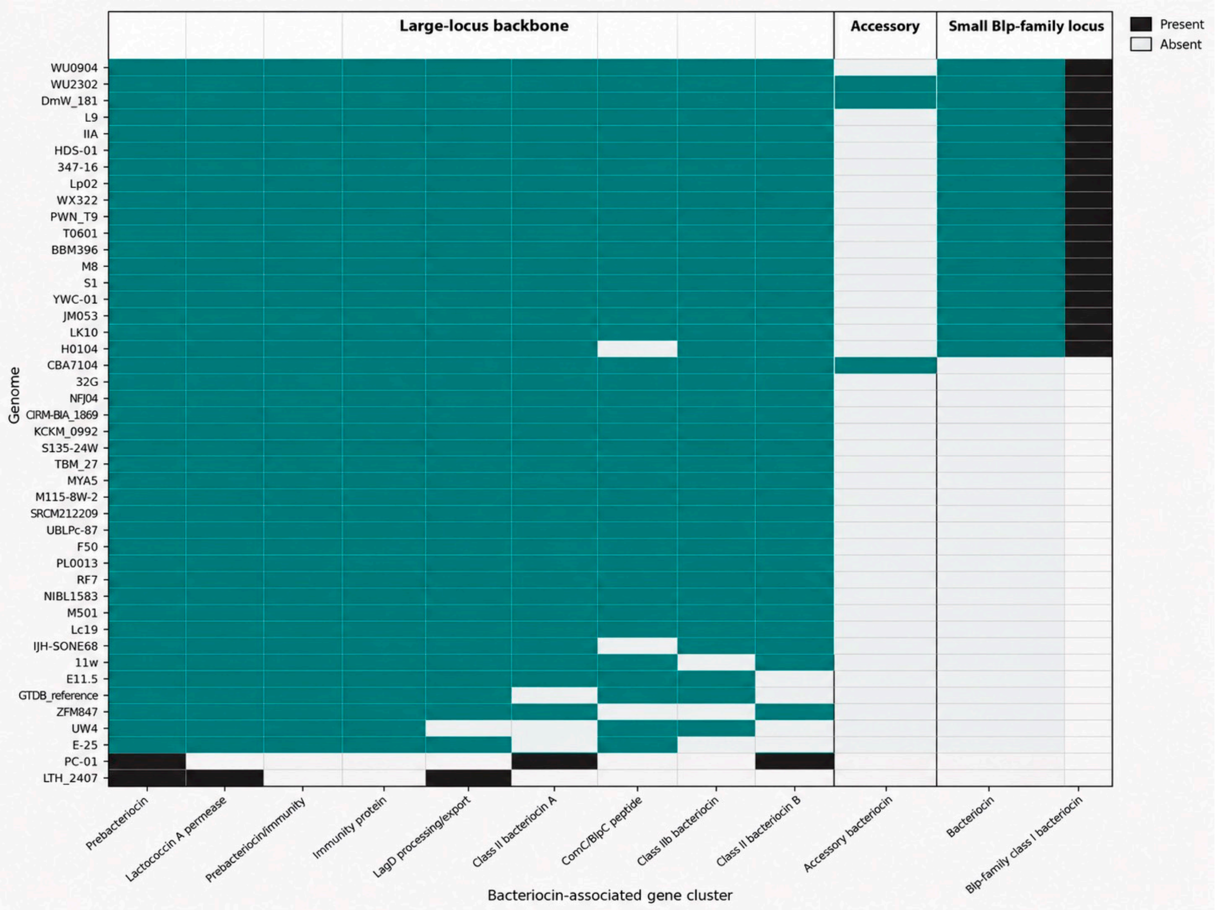

Supplementary Figure S1. Distribution of bacteriocin-associated loci across the 44-genome *Lacticaseibacillus paracasei* dataset. Presence/absence patterns are shown for the nine-component large-locus backbone, the two-gene small Blp-family locus, and the group_2723 accessory bacteriocin candidate across the 44 genomes.

PDMMEO_01283 (WU0904) & ECDLDI_00177 (WU2302)

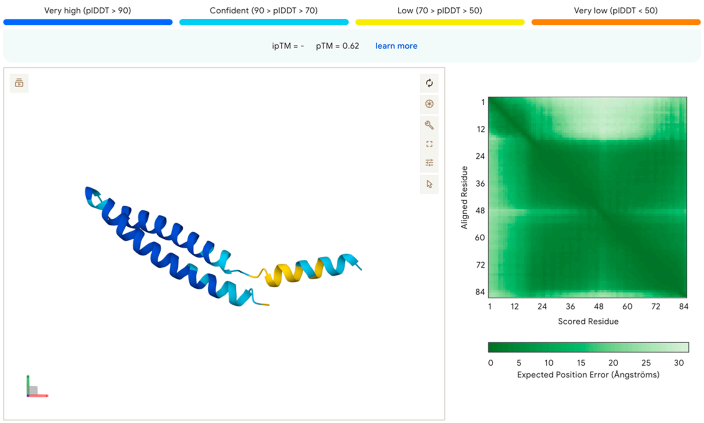

PDMMEO 00357 (WU0904) & ECDLDI 01460

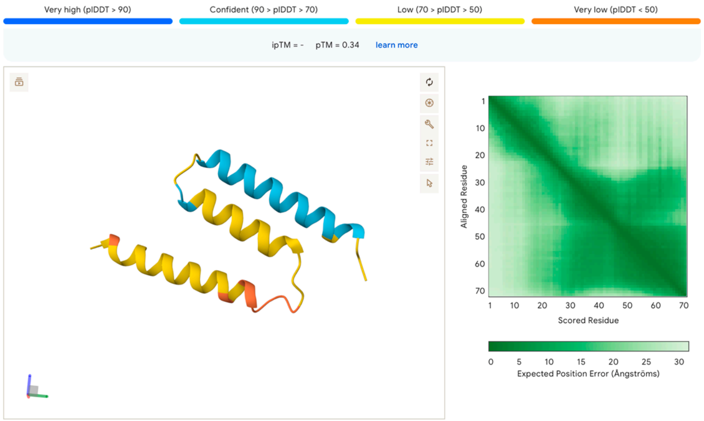

ECDLDI 01475 (WU2302)

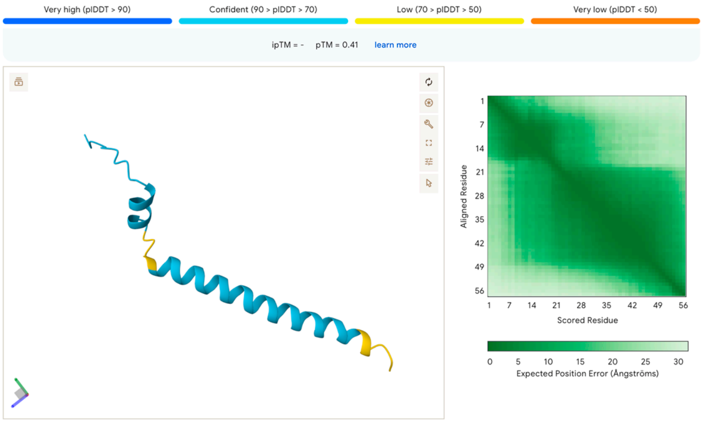

Supplementary Figure S2. The predicted TM-score, a confidence metric that AlphaFold outputs alongside each predicted structure
