# Supplementary material for: Comparative Genomics Reveals Conserved Bacteriocin-Associated Loci in Lacticaseibacillus paracasei Strains with Anti-MRSA Activity Isolated from Palmyra Palm Sugar
Source: Antibiotics (Basel). 2026 Oct 9;15(10):1000. doi: 10.3390/antibiotics15101000 (PMC13700875; doi:10.3390/antibiotics15101000)
Supplement: Supplementary file 1 [file antibiotics-15-01000-s001.zip › 20260928 Supplementary table.pdf]

Table S1. Audit of the preliminary 66-genome collection, including accessions, niche classifications, inclusion status, and exclusion reasons.

sample_id	assembly_accession	species_label	niche_group	source	strain	isolation_source	host	geo_location	genome_filename	inclusion_status	exclusion_reason
WU0904	WU0904_local	Lacticaseibacillus sp. local isolate	local_isolate	local	WU0904				WU0904_local	included_in_final44	retained
WU2302	WU2302_local	Lacticaseibacillus sp. local isolate	local_isolate	local	WU2302				WU2302_local	included_in_final44	retained
GCF_000615205.1	GCF_000615205.1	Lacticaseibacillus casei DSM 20011 = JCM	E_human_or_gut	outgroup_type_material	JCM 1134				GCF_000615205.1__JCM_1134	excluded_before_final44	failed_species_filter_non_paracasei_ANI_below_95_to_GCF_000829035.1_reference

sample_id	assembly_accession	species_label	niche_group	source	strain	isolation_source	host	geo_location	genome_filename	inclusion_status	exclusion_reason
		1134 = ATCC 393									
GCF_000829055.1	GCF_000829055.1	Lacticaseibacillus casei DSM 20011 = JCM 1134 = ATCC 393	H_unknown	outgroup_type_material	ATCC 393				GCF_000829055.1__ATCC_393	excluded_before_final44	failed_species_filter_non_paracasei_ANI_below_95_to_GCF_000829035.1_reference
GCF_002849515.1	GCF_002849515.1	Lacticaseibacillus rhamnosus	H_unknown	outgroup_type_material	NRRL B-442	missing		USA	GCF_002849515.1__NRRL_B-442	excluded_before_final44	failed_species_filter_non_paracasei_ANI_below_95_to_GCF_000829035.1_reference
GCF_9006369	GCF_9006369	Lacticaseibacillu	H_unknown	outgroup_type_m	NCTC1	not available: to	not avail		GCF_900636965.1__NCTC 13764	excluded_before_	failed_species_filter_non_paracasei_ANI_below_95_to_GCF_000829035.1_reference

sample_id	assembly_accession	species_label	niche_group	source	strain	isolation_source	host	geo_location	genome_filename	inclusion_status	exclusion_reason
65.1	65.1	s rhamnosus		aterial	3764	be reported later	able			final44	
GCF_000309605.1	GCF_000309605.1	Lacticaseibacillus paracasei	B_fermented_plant_or_vegetable	public_ncbi	32G	corn silage		USA: Wisconsin	GCF_000309605.1__32G	included_in_final44	retained
GCF_000376145.1	GCF_000376145.1	Lacticaseibacillus paracasei	G_environment	public_ncbi	UW4			Missing	GCF_000376145.1__UW4	included_in_final44	retained
GCF_001244395.1	GCF_001244395.1	Lacticaseibacillus paracasei	E_human_or_gut	public_ncbi	L9	isolated from the feces of a centenarian	Homo sapiens	China: village Bama, guangxi	GCF_001244395.1__L9	included_in_final44	retained

sample_id	assembly_accession	species_label	niche_group	source	strain	isolation_source	host	geo_location	genome_filename	inclusion_status	exclusion_reason
GCF_002079285.1	GCF_002079285.1	Lacticaseibacillus paracasei	F_animal	public_ncbi	IIA	beef from cattle Peranakan Ongole		Indonesia: Bogor	GCF_002079285.1__IIA	included_in_final44	retained
GCF_002115655.1	GCF_002115655.1	Lacticaseibacillus paracasei	B_fermented_plant_or_vegetable	public_ncbi	DmW_181		Drosophila	USA: New York	GCF_002115655.1__DmW_181	included_in_final44	retained
GCF_002865565.1	GCF_002865565.1	Lacticaseibacillus paracasei	B_fermented_plant_or_vegetable	public_ncbi	HD1.7	Chinese sauerkraut	missing	China:Heilongjiang provience	GCF_002865565.1__HD1.7	excluded_before_final44	dereplicated_to_GCF_002902825.1__HDS-01
GCF_002902825.1	GCF_002902825.1	Lacticaseibacillus paracasei	B_fermented_plant_or_vegetable	public_ncbi	HDS-01	Chinese sauerkraut	missing	China:Heilongjiang provience	GCF_002902825.1__HDS-01	included_in_final44	retained

sample_id	assembly_accession	species_label	niche_group	source	strain	isolation_source	host	geo_location	genome_filename	inclusion_status	exclusion_reason
GCF_003966835.1	GCF_003966835.1	Lacticaseibacillus paracasei	B_fermented_plant_or_vegetable	public_ncbi	IJH-SONE68			Japan:Fukuoka	GCF_003966835.1__IJH-SONE68	included_in_final44	retained
GCF_007292115.1	GCF_007292115.1	Lacticaseibacillus paracasei	B_fermented_plant_or_vegetable	public_ncbi	CBA3611	human-fecal	Homo sapiens	South Korea: Gwangju	GCF_007292115.1__CBA3611	excluded_before_final44	dereplicated_to_GCF_058070825.1__Lc19
GCF_009931715.1	GCF_009931715.1	Lacticaseibacillus paracasei	E_human_or_gut	public_ncbi	CACC566	Canine feces	Canine	South Korea:Jeongeup-si	GCF_009931715.1__CACC_566	excluded_before_final44	dereplicated_to_GCF_012955485.1__347-16
GCF_012955485.1	GCF_012955485.1	Lacticaseibacillus paracasei	E_human_or_gut	public_ncbi	347-16	feces	Homo sapiens	Russia: Kislovodsk	GCF_012955485.1__347-16	included_in_final44	retained
GCF_0133071	GCF_0133071	Lacticaseibac	E_human_or_gut	public_ncbi	Lp02	feces	Homo	South Korea:Ky	GCF_0133071 25.1__Lp02	included_in_fi	retained

sample_id	assembly_accession	species_label	niche_group	source	strain	isolation_source	host	geo_location	genome_filename	inclusion_status	exclusion_reason
25.1	25.1	illus paracasei					sapiens	unggi-do		nal44	
GCF_014905075.1	GCF_014905075.1	Lacticaseibacillus paracasei	F_animal	public_ncbi	NFFJ04	Animal feed		South Korea: Sangju	GCF_014905075.1__NFFJ04	included_in_final44	retained
GCF_022701315.1	GCF_022701315.1	Lacticaseibacillus paracasei	B_fermented_plant_or_vegetable	public_ncbi	WX322		Pickle	China: Chongqing	GCF_022701315.1__WX322	included_in_final44	retained
GCF_025190485.1	GCF_025190485.1	Lacticaseibacillus paracasei	A_palm_sap_or_palm_sugar	public_ncbi	CIRM-BIA1869	birch sap		France	GCF_025190485.1__CIRM-BIA_1869	included_in_final44	retained
GCF_028613785.1	GCF_028613785.1	Lacticaseibacillus par	B_fermented_plant_or_vegetable	public_ncbi	H0104	vegetable		China: Yunnan	GCF_028613785.1__H0104	included_in_final44	retained

sample_id	assembly_accession	species_label	niche_group	source	strain	isolation_source	host	geo_location	genome_filename	inclusion_status	exclusion_reason
		acasei									
GCF_030418055.1	GCF_030418055.1	Lacticaseibacillus paracasei	A_palm_sap_or_palm_sugar	public_ncbi	PWN_T9	Palm wine	Elaeis guineensis	Nigeria: Lagos	GCF_030418055.1__PWN_T9	included_in_final44	retained
GCF_030540905.1	GCF_030540905.1	Lacticaseibacillus paracasei	B_fermented_plant_or_vegetable	public_ncbi	11w	fermented pear		Lithuania: Vilnius	GCF_030540905.1__11w	included_in_final44	retained
GCF_031348425.1	GCF_031348425.1	Lacticaseibacillus paracasei	B_fermented_plant_or_vegetable	public_ncbi	KCKM0245	kimchi		South Korea	GCF_031348425.1__KCKM_0245	excluded_before_final44	dereplicated_to_GCF_031461095.1__KCKM_0992
GCF_031461095.1	GCF_031461095.1	Lacticaseibacillus paracasei	B_fermented_plant_or_vegetable	public_ncbi	KCKM0992	kimchi		South Korea	GCF_031461095.1__KCKM_0992	included_in_final44	retained

sample_id	assembly_accession	species_label	niche_group	source	strain	isolation_source	host	geo_location	genome_filename	inclusion_status	exclusion_reason
GCF_035330705.1	GCF_035330705.1	Lacticaseibacillus paracasei	E_human_or_gut	public_ncbi	S135-24W	Infant feces	Homo sapiens	Taiwan	GCF_035330705.1__S135-24W	included_in_final44	retained
GCF_035586595.1	GCF_035586595.1	Lacticaseibacillus paracasei	B_fermented_plant_or_vegetable	public_ncbi	HP-B1337			China	GCF_035586595.1__HP-B1337	excluded_before_final44	dereplicated_to_GCF_057978265.1__M501
GCF_039615415.1	GCF_039615415.1	Lacticaseibacillus paracasei	B_fermented_plant_or_vegetable	public_ncbi	TBM27	fermented soybean		Viet Nam: Bac Giang	GCF_039615415.1__TBM_27	included_in_final44	retained
GCF_040459555.1	GCF_040459555.1	Lacticaseibacillus paracasei	A_palm_sap_or_palm_sugar	public_ncbi	T1901	fermented palm sap		Thailand	GCF_040459555.1__T1901	excluded_before_final44	dereplicated_to_WU0904_local
GCF_040459595	GCF_040459595	Lacticaseibac	A_palm_sap_or_pa	public_ncbi	T1301	fermented		Thailand	GCF_040459585.1__T1301	excluded_before_	dereplicated_to_WU0904_local

sample_id	assembly_accession	species_label	niche_group	source	strain	isolation_source	host	geo_location	genome_filename	inclusion_status	exclusion_reason
85.1	85.1	illus paracasei	lm_sugar			palm sap				final44	
GCF_040459615.1	GCF_040459615.1	Lacticaseibacillus paracasei	A_palm_sap_or_palm_sugar	public_ncbi	T1304	fermented palm sap		Thailand	GCF_040459615.1__T1304	excluded_before_final44	dereplicated_to_WU0904_local
GCF_040459635.1	GCF_040459635.1	Lacticaseibacillus paracasei	A_palm_sap_or_palm_sugar	public_ncbi	T0602	fermented palm sap		Thailand	GCF_040459635.1__T0602	excluded_before_final44	dereplicated_to_GCF_040459685.1__T0601
GCF_040459675.1	GCF_040459675.1	Lacticaseibacillus paracasei	A_palm_sap_or_palm_sugar	public_ncbi	T0902	fermented palm sap		Thailand	GCF_040459675.1__T0902	excluded_before_final44	dereplicated_to_WU0904_local
GCF_040459685.1	GCF_040459685.1	Lacticaseibacillus par	A_palm_sap_or_palm_sugar	public_ncbi	T0601	fermented palm sap		Thailand	GCF_040459685.1__T0601	included_in_final44	retained

sample_id	assembly_accession	species_label	niche_group	source	strain	isolation_source	host	geo_location	genome_filename	inclusion_status	exclusion_reason
		acasei									
GCF_041117985.1	GCF_041117985.1	Lacticaseibacillus paracasei	B_fermented_plant_or_vegetable	public_ncbi	MYA5	kimchi	missing	South Korea: Gwangju	GCF_041117985.1__MYA5	included_in_final44	retained
GCF_041734755.1	GCF_041734755.1	Lacticaseibacillus paracasei	D_dairy	public_ncbi	YGRT25	yogurt		South Korea	GCF_041734755.1__YGRT25	excluded_before_final44	dereplicated_to_GCF_058070825.1__Lc19
GCF_044740695.1	GCF_044740695.1	Lacticaseibacillus paracasei	D_dairy	public_ncbi	E11.5	Dairy product		Ethiopia	GCF_044740695.1__E11.5	included_in_final44	retained
GCF_045256935.1	GCF_045256935.1	Lacticaseibacillus paracasei	D_dairy	public_ncbi	Egylham_14	raw camel milk	Camelus dromedarius	Egypt	GCF_045256935.1__Egylham_14	excluded_before_final44	dereplicated_to_GCF_048500655.1__CBA7104

sample_id	assembly_accession	species_label	niche_group	source	strain	isolation_source	host	geo_location	genome_filename	inclusion_status	exclusion_reason
GCF_046116425.1	GCF_046116425.1	Lacticaseibacillus paracasei	D_dairy	public_ncbi	M115-8W-2	milk	Homo sapiens	Taiwan	GCF_046116425.1__M115-8W-2	included_in_final44	retained
GCF_046581425.1	GCF_046581425.1	Lacticaseibacillus paracasei	E_human_or_gut	public_ncbi	BBM398	the feces of healthy humans	not provided	Taiwan:Taipei	GCF_046581425.1__BBM398	excluded_before_final44	dereplicated_to_GCF_046581445.1__BBM396
GCF_046581435.1	GCF_046581435.1	Lacticaseibacillus paracasei	E_human_or_gut	public_ncbi	BBM397	the feces of healthy humans	not provided	Taiwan:Taipei	GCF_046581435.1__BBM397	excluded_before_final44	dereplicated_to_GCF_046581445.1__BBM396
GCF_046581445.1	GCF_046581445.1	Lacticaseibacillus paracasei	E_human_or_gut	public_ncbi	BBM396	the feces of healthy humans	not provided	Taiwan:Taipei	GCF_046581445.1__BBM396	included_in_final44	retained

sample_id	assembly_accession	species_label	niche_group	source	strain	isolation_source	host	geo_location	genome_filename	inclusion_status	exclusion_reason
GCF_047785155.1	GCF_047785155.1	Lacticaseibacillus paracasei	B_fermented_plant_or_vegetable	public_ncbi	SRCM212209	kimchi		South Korea:Sunchang	GCF_047785155.1__SRCM212209	included_in_final44	retained
GCF_048500655.1	GCF_048500655.1	Lacticaseibacillus paracasei	E_human_or_gut	public_ncbi	CBA7104	adult feces	Homo sapiens	South Korea: Bundang	GCF_048500655.1__CBA7104	included_in_final44	retained
GCF_049201625.1	GCF_049201625.1	Lacticaseibacillus paracasei	B_fermented_plant_or_vegetable	public_ncbi	UBLPc-87	Fermented vegetable		India: Hyderabad	GCF_049201625.1__UBLPc-87	included_in_final44	retained
GCF_049532865.1	GCF_049532865.1	Lacticaseibacillus paracasei	D_dairy	public_ncbi	PC-01	Fermented yak milk		China:Lhasa	GCF_049532865.1__PC-01	included_in_final44	retained
GCF_0503202	GCF_0503202	Lacticaseibac	C_other_fermente	public_ncbi	LTH2407	Gouda		Germany	GCF_050320215.1__	included_in_fi	retained

sample_id	assembly_accession	species_label	niche_group	source	strain	isolation_source	host	geo_location	genome_filename	inclusion_status	exclusion_reason
15.1	15.1	illus paracasei	d_food			cheese			LTH_2407	nal44	
GCF_050596945.1	GCF_050596945.1	Lacticaseibacillus paracasei	E_human_or_gut	public_ncbi	CYLB1019	Cheng Kung University Hospital-stool sample	Homo sapiens	Taiwan	GCF_050596945.1__CYLB_1019	excluded_before_final44	dereplicated_to_GCF_058070825.1__Lc19
GCF_051123965.1	GCF_051123965.1	Lacticaseibacillus paracasei	D_dairy	public_ncbi	M8	fermented tofu whey		China:GuangDong	GCF_051123965.1__M8	included_in_final44	retained
GCF_051514555.1	GCF_051514555.1	Lacticaseibacillus paracasei	D_dairy	public_ncbi	ZFM847	fresh milk		China	GCF_051514555.1__ZFM847	included_in_final44	retained

sample_id	assembly_accession	species_label	niche_group	source	strain	isolation_source	host	geo_location	genome_filename	inclusion_status	exclusion_reason
GCF_052588795.2	GCF_052588795.2	Lacticaseibacillus paracasei	A_palm_sap_or_palm_sugar	public_ncbi	S1	sap	Nypa fruticans	Philippines:Ilocos Norte	GCF_052588795.2__S1	included_in_final44	retained
GCF_052601095.1	GCF_052601095.1	Lacticaseibacillus paracasei	C_other_fermented_food	public_ncbi	YWC-01	fermented food		China: Shandong	GCF_052601095.1__YWC-01	included_in_final44	retained
GCF_052904645.1	GCF_052904645.1	Lacticaseibacillus paracasei	B_fermented_plant_or_vegetable	public_ncbi	F50			China: ChongQing	GCF_052904645.1__F50	included_in_final44	retained
GCF_052907405.1	GCF_052907405.1	Lacticaseibacillus paracasei	B_fermented_plant_or_vegetable	public_ncbi	NCU215	fermented vegetable		China: Jiangxi Nanchang	GCF_052907405.1__NCU215	excluded_before_final44	dereplicated_to_GCF_057978265.1__M501
GCF_053749055.1	GCF_053749055.1	Lacticaseibacillus bac	B_fermented_plant_or_v	public_ncbi	PL0013	kimchi		Philippines	GCF_053749055.1__	included_in_fi	retained

sample_id	assembly_accession	species_label	niche_group	source	strain	isolation_source	host	geo_location	genome_filename	inclusion_status	exclusion_reason
55.1	55.1	illus paracasei	egetable						PL0013	nal44	
GCF_054570615.1	GCF_054570615.1	Lacticaseibacillus paracasei	B_fermented_plant_or_vegetable	public_ncbi	LPTSRWCO1		Isolated from Soaked rice water extraxt	India: Tamil Nadu, Coimbatore,Thondamuthur, Latitude, 10.947071, Longitude, 76.746457	GCF_054570615.1__LPTSRWCO1	excluded_before_final44	dereplicated_to_GCF_058070825.1__Lc19
GCF_055597705.1	GCF_055597705.1	Lacticaseibacillus paracasei	F_animal	public_ncbi	E-25		Bos taurus	Russia: Valaam, Republic of Karelia	GCF_055597705.1__E-25	included_in_final44	retained
GCF_055597745.1	GCF_055597745.1	Lacticaseibacillus par	F_animal	public_ncbi	E-49		Bos taurus	Russia: Valaam, Republic of	GCF_055597745.1__E-49	excluded_before_final44	dereplicated_to_GCF_058070825.1__Lc19

sample_id	assembly_accession	species_label	niche_group	source	strain	isolation_source	host	geo_location	genome_filename	inclusion_status	exclusion_reason
		acasei						Karelia			
GCF_055699145.1	GCF_055699145.1	Lacticaseibacillus paracasei	C_other_fermented_food	public_ncbi	RF7	Kefir		China: Kuitun, Xinjiang	GCF_055699145.1__RF7	included_in_final44	retained
GCF_056619785.1	GCF_056619785.1	Lacticaseibacillus paracasei	D_dairy	public_ncbi	JM053	Fermented dairy product		China:Guangdong	GCF_056619785.1__JM053	included_in_final44	retained
GCF_056665955.1	GCF_056665955.1	Lacticaseibacillus paracasei	B_fermented_plant_or_vegetable	public_ncbi	NIBL1583	kimchi		South Korea	GCF_056665955.1__NIBL1583	included_in_final44	retained
GCF_057418975.1	GCF_057418975.1	Lacticaseibacillus paracasei	B_fermented_plant_or_vegetable	public_ncbi	LK10		soybean albumin ferm	China:Hunan	GCF_057418975.1__LK10	included_in_final44	retained

sample_id	assembly_accession	species_label	niche_group	source	strain	isolation_source	host	geo_location	genome_filename	inclusion_status	exclusion_reason
							entation liquor				
GCF_057978265.1	GCF_057978265.1	Lacticaseibacillus paracasei	D_dairy	public_ncbi	M501	milk		China: QingDao	GCF_057978265.1__M501	included_in_final44	retained
GCF_058070825.1	GCF_058070825.1	Lacticaseibacillus paracasei	D_dairy	public_ncbi	Lc19	fecal sample	Homo sapiens	China: Guangxi, bama County	GCF_058070825.1__Lc19	included_in_final44	retained
GCF_058134255.1	GCF_058134255.1	Lacticaseibacillus paracasei	A_palm_sap_or_palm_sugar	public_ncbi	WU2502			Thailand	GCF_058134255.1__WU2502	excluded_before_final44	dereplicated_to_WU2302_local

- Table **S2a Assembly quality information for the final dereplicated genome collection.**

sample_id	num_contigs	largest_contig_bp	genome_size_bp	gc_percent	n50_bp	l50	Ns_per_100kb	quality_flag
GCF_001244395.1__L9	1	3076437	3076437	46.35	3076437	1	0.03	Pass
GCF_002902825.1__HDS-01	1	3038767	3038767	46.42	3038767	1	0	Pass
GCF_035330705.1__S135-24W	1	3116723	3116723	46.37	3116723	1	0	Pass
GCF_049532865.1__PC-01	1	2829327	2829327	46.64	2829327	1	0	Pass
GCF_046581445.1__BBM396	2	2939216	3014751	46.29	2939216	1	0	Pass
GCF_052601095.1__YWC-01	2	2927251	2984031	46.53	2927251	1	0	Pass
GCF_056619785.1__JM053	2	3136444	3139540	46.32	3136444	1	0	Pass
GCF_057978265.1__M501	2	3102303	3151054	46.33	3102303	1	0	Pass
GCF_058070825.1__Lc19	2	3075217	3140886	46.28	3075217	1	0	Pass
GCF_000829035.1__GTDB_reference	3	2995875	3017804	46.55	2995875	1	0	Pass
GCF_003966835.1__IJH-SONE68	3	3084917	3181204	46.39	3084917	1	0	Pass
GCF_012955485.1__347-16	3	3102350	3219033	46.14	3102350	1	0	Pass
GCF_013307125.1__Lp02	3	2991176	3092262	46.33	2991176	1	0	Pass

GCF_046116425.1__M115-8W-2	3	2898150	2957992	46.34	2898150	1	0	Pass
GCF_048500655.1__CBA7104	3	3063928	3187641	46.19	3063928	1	0	Pass
GCF_051123965.1__M8	3	3258331	3293134	46.41	3258331	1	0	Pass
GCF_052904645.1__F50	3	3067873	3119264	46.39	3067873	1	0	Pass
GCF_056665955.1__NIBL1583	3	3100882	3151564	46.27	3100882	1	0	Pass
GCF_002079285.1__IIA	4	3055892	3246143	46.25	3055892	1	0	Pass
GCF_014905075.1__NFFJ04	4	2983945	3196149	46.3	2983945	1	0	Pass
GCF_041117985.1__MYA5	4	3059220	3144787	46.29	3059220	1	0	Pass
GCF_057418975.1__LK10	4	3003560	3136649	46.45	3003560	1	0	Pass
GCF_044740695.1__E11.5	5	3063221	3190861	46.37	3063221	1	0	Pass
GCF_050320215.1__LTH_2407	5	2715484	2900557	46.48	2715484	1	0	Pass
GCF_051514555.1__ZFM847	5	2995448	3139050	46.36	2995448	1	0	Pass
GCF_055699145.1__RF7	6	2833284	3081250	46.21	2833284	1	0	Pass
GCF_031461095.1__KCKM_0992	7	3042295	3267117	46.21	3042295	1	0	Pass
GCF_047785155.1__SRCM212209	8	2987076	3300554	46.26	2987076	1	0	Pass

GCF_022701315.1__WX322	9	3080525	3320114	46.12	3080525	1	0	Pass
GCF_030418055.1__PWN_T9	18	528992	3007208	46.3	289765	4	16.63	Pass
GCF_052588795.2__S1	41	651286	3056183	46.12	350017	3	13.09	Pass
GCF_000309605.1__32G	42	333321	3011496	46.37	172346	7	7.24	Pass
GCF_053749055.1__PL0013	70	276848	2860317	46.36	131010	8	0	Pass
GCF_040459685.1__T0601	84	417502	3072098	46.14	158110	5	25.06	Pass
GCF_025190485.1__CIRM-BIA_1869	103	460967	3062932	46.18	125620	7	0	Pass
GCF_002115655.1__DmW_181	127	515814	3200973	46.14	72562	13	0	Pass
WU2302_local	130	330839	3024432	46.27	126405	9	6.41	Pass
GCF_039615415.1__TBM_27	132	213059	3106278	46.21	58121	15	0	Pass
GCF_030540905.1__11w	135	228441	3140922	46.07	84292	11	29.13	Pass
GCF_000376145.1__UW4	144	170908	2628832	46.41	41179	17	1.03	Pass
GCF_049201625.1__UBLPc-87	175	238316	2947161	46.31	143953	8	1.7	Pass_with_warning
GCF_055597705.1__E-25	187	208894	2882254	46.44	59137	14	0	Pass_with_warning
GCF_028613785.1__H0104	189	166519	3154462	46.21	71531	15	5.14	Pass_with_warning
WU0904_local	215	437549	3128132	46.11	166913	6	18.54	Pass_with_warning

Table S2b. Selected metadata information for the final dereplicated genome collection.

sample_id	quality_score	ANI_to_reference_percent	AF_sample_to_reference_percent	AF_reference_to_sample_percent
GCF_000309605.1__32G	100.00	98.61	82.77	82.60
GCF_000376145.1__UW4	100.00	98.53	87.87	76.54
GCF_000829035.1__GTDB_reference	5100.00	100.00	100.00	100.00
GCF_001244395.1__L9	100.00	98.27	81.36	82.94
GCF_002079285.1__IIA	100.00	98.29	77.44	83.29
GCF_002115655.1__DmW_181	100.00	98.21	77.91	82.64
GCF_002902825.1__HDS-01	100.00	98.25	81.41	81.98
GCF_003966835.1__IJH-SONE68	100.00	98.20	79.11	83.40
GCF_012955485.1__347-16	100.00	98.27	77.23	82.38
GCF_013307125.1__Lp02	100.00	98.27	78.59	80.53
GCF_014905075.1__NFFJ04	100.00	98.30	77.66	82.25
GCF_022701315.1__WX322	100.00	98.26	76.48	84.14
GCF_025190485.1__CIRM-BIA_1869	100.00	98.38	81.63	82.85
GCF_028613785.1__H0104	100.00	98.49	78.42	81.97
GCF_030418055.1__PWN_T9	100.00	98.43	81.82	81.53
GCF_030540905.1__11w	100.00	98.42	78.97	82.20
GCF_031461095.1__KCKM_0992	100.00	98.32	77.81	84.24
GCF_035330705.1__S135-24W	100.00	98.32	81.06	83.72
GCF_039615415.1__TBM_27	100.00	98.47	80.71	83.08

GCF_040459685.1__ T0601	100.00	98.26	81.05	82.51
GCF_041117985.1__ MYA5	100.00	98.00	80.91	84.31
GCF_044740695.1__ E11.5	100.00	98.46	80.05	84.65
GCF_046116425.1__ M115-8W-2	100.00	98.21	85.32	83.63
GCF_046581445.1__ BBM396	100.00	97.98	82.01	81.93
GCF_047785155.1__ SRCM212209	100.00	98.36	76.46	83.62
GCF_048500655.1__ CBA7104	100.00	98.35	80.17	84.69
GCF_049201625.1__ UBLPc-87	100.00	98.48	85.30	83.31
GCF_049532865.1__ PC-01	100.00	98.27	83.12	77.93
GCF_050320215.1__ LTH_2407	100.00	98.19	82.31	79.11
GCF_051123965.1__ M8	100.00	98.24	76.66	83.65
GCF_051514555.1__ ZFM847	100.00	98.24	79.89	83.10
GCF_052588795.2__ S1	100.00	98.46	80.99	82.02
GCF_052601095.1__ YWC-01	100.00	98.34	83.66	82.73
GCF_052904645.1__ F50	100.00	98.25	82.05	84.81
GCF_053749055.1__ PL0013	100.00	98.36	85.75	81.28
GCF_055597705.1__ E-25	100.00	98.90	84.32	80.53
GCF_055699145.1__ RF7	100.00	98.30	80.46	82.15
GCF_056619785.1__ JM053	100.00	98.27	80.21	83.44
GCF_056665955.1__ NIBL1583	100.00	98.26	78.70	82.19
GCF_057418975.1__ LK10	100.00	98.41	80.58	83.75
GCF_057978265.1__ M501	100.00	98.36	81.15	84.74

GCF_058070825.1__Lc19	100.00	98.35	79.96	83.22
WU0904_local	100000.00	98.47	79.71	82.62
WU2302_local	100000.00	98.24	82.75	82.94

Table S2c. taxonomy/ANI information for the final dereplicated genome collection.

**sample_id**	**GTDB_species**	**taxonomy_comment**
WU0904_local		GTDB-Tk and ANI software were unavailable in this run, so the local genomes cannot be assigned confidently from genome-wide taxonomy here.
WU2302_local		GTDB-Tk and ANI software were unavailable in this run, so the local genomes cannot be assigned confidently from genome-wide taxonomy here.
GCF_000615205.1	Lacticaseibacillus casei	No genome-wide ANI available; species label is carried from input metadata only.
GCF_000829055.1	Lacticaseibacillus casei	No genome-wide ANI available; species label is carried from input metadata only.

GCF_002849515.1	Lacticaseibacillus rhamnosus	No genome-wide ANI available; species label is carried from input metadata only.
GCF_900636965.1	Lacticaseibacillus rhamnosus	No genome-wide ANI available; species label is carried from input metadata only.
GCF_000309605.1	Lacticaseibacillus paracasei	No genome-wide ANI available; species label is carried from input metadata only.
GCF_000376145.1	Lacticaseibacillus paracasei	No genome-wide ANI available; species label is carried from input metadata only.
GCF_001244395.1	Lacticaseibacillus paracasei	No genome-wide ANI available; species label is carried from input metadata only.
GCF_002079285.1	Lacticaseibacillus paracasei	No genome-wide ANI available; species label is carried from input metadata only.
GCF_002115655.1	Lacticaseibacillus paracasei	No genome-wide ANI available; species label is carried from input metadata only.
GCF_002865565.1	Lacticaseibacillus paracasei	No genome-wide ANI available; species label is carried from input metadata only.
GCF_002902825.1	Lacticaseibacillus paracasei	No genome-wide ANI available; species label is carried from input metadata only.

GCF_003966835.1	Lacticaseibacillus paracasei	No genome-wide ANI available; species label is carried from input metadata only.
GCF_007292115.1	Lacticaseibacillus paracasei	No genome-wide ANI available; species label is carried from input metadata only.
GCF_009931715.1	Lacticaseibacillus paracasei	No genome-wide ANI available; species label is carried from input metadata only.
GCF_012955485.1	Lacticaseibacillus paracasei	No genome-wide ANI available; species label is carried from input metadata only.
GCF_013307125.1	Lacticaseibacillus paracasei	No genome-wide ANI available; species label is carried from input metadata only.
GCF_014905075.1	Lacticaseibacillus paracasei	No genome-wide ANI available; species label is carried from input metadata only.
GCF_022701315.1	Lacticaseibacillus paracasei	No genome-wide ANI available; species label is carried from input metadata only.
GCF_025190485.1	Lacticaseibacillus paracasei	No genome-wide ANI available; species label is carried from input metadata only.
GCF_028613785.1	Lacticaseibacillus paracasei	No genome-wide ANI available; species label is carried from input metadata only.

GCF_030418055.1	Lacticaseibacillus paracasei	No genome-wide ANI available; species label is carried from input metadata only.
GCF_030540905.1	Lacticaseibacillus paracasei	No genome-wide ANI available; species label is carried from input metadata only.
GCF_031348425.1	Lacticaseibacillus paracasei	No genome-wide ANI available; species label is carried from input metadata only.
GCF_031461095.1	Lacticaseibacillus paracasei	No genome-wide ANI available; species label is carried from input metadata only.
GCF_035330705.1	Lacticaseibacillus paracasei	No genome-wide ANI available; species label is carried from input metadata only.
GCF_035586595.1	Lacticaseibacillus paracasei	No genome-wide ANI available; species label is carried from input metadata only.
GCF_039615415.1	Lacticaseibacillus paracasei	No genome-wide ANI available; species label is carried from input metadata only.
GCF_040459555.1	Lacticaseibacillus paracasei	No genome-wide ANI available; species label is carried from input metadata only.
GCF_040459585.1	Lacticaseibacillus paracasei	No genome-wide ANI available; species label is carried from input metadata only.

GCF_040459615.1	Lacticaseibacillus paracasei	No genome-wide ANI available; species label is carried from input metadata only.
GCF_040459635.1	Lacticaseibacillus paracasei	No genome-wide ANI available; species label is carried from input metadata only.
GCF_040459675.1	Lacticaseibacillus paracasei	No genome-wide ANI available; species label is carried from input metadata only.
GCF_040459685.1	Lacticaseibacillus paracasei	No genome-wide ANI available; species label is carried from input metadata only.
GCF_041117985.1	Lacticaseibacillus paracasei	No genome-wide ANI available; species label is carried from input metadata only.
GCF_041734755.1	Lacticaseibacillus paracasei	No genome-wide ANI available; species label is carried from input metadata only.
GCF_044740695.1	Lacticaseibacillus paracasei	No genome-wide ANI available; species label is carried from input metadata only.
GCF_045256935.1	Lacticaseibacillus paracasei	No genome-wide ANI available; species label is carried from input metadata only.
GCF_046116425.1	Lacticaseibacillus paracasei	No genome-wide ANI available; species label is carried from input metadata only.

GCF_046581425.1	Lacticaseibacillus paracasei	No genome-wide ANI available; species label is carried from input metadata only.
GCF_046581435.1	Lacticaseibacillus paracasei	No genome-wide ANI available; species label is carried from input metadata only.
GCF_046581445.1	Lacticaseibacillus paracasei	No genome-wide ANI available; species label is carried from input metadata only.
GCF_047785155.1	Lacticaseibacillus paracasei	No genome-wide ANI available; species label is carried from input metadata only.
GCF_048500655.1	Lacticaseibacillus paracasei	No genome-wide ANI available; species label is carried from input metadata only.
GCF_049201625.1	Lacticaseibacillus paracasei	No genome-wide ANI available; species label is carried from input metadata only.
GCF_049532865.1	Lacticaseibacillus paracasei	No genome-wide ANI available; species label is carried from input metadata only.
GCF_050320215.1	Lacticaseibacillus paracasei	No genome-wide ANI available; species label is carried from input metadata only.
GCF_050596945.1	Lacticaseibacillus paracasei	No genome-wide ANI available; species label is carried from input metadata only.

GCF_051123965.1	Lacticaseibacillus paracasei	No genome-wide ANI available; species label is carried from input metadata only.
GCF_051514555.1	Lacticaseibacillus paracasei	No genome-wide ANI available; species label is carried from input metadata only.
GCF_052588795.2	Lacticaseibacillus paracasei	No genome-wide ANI available; species label is carried from input metadata only.
GCF_052601095.1	Lacticaseibacillus paracasei	No genome-wide ANI available; species label is carried from input metadata only.
GCF_052904645.1	Lacticaseibacillus paracasei	No genome-wide ANI available; species label is carried from input metadata only.
GCF_052907405.1	Lacticaseibacillus paracasei	No genome-wide ANI available; species label is carried from input metadata only.
GCF_053749055.1	Lacticaseibacillus paracasei	No genome-wide ANI available; species label is carried from input metadata only.
GCF_054570615.1	Lacticaseibacillus paracasei	No genome-wide ANI available; species label is carried from input metadata only.
GCF_055597705.1	Lacticaseibacillus paracasei	No genome-wide ANI available; species label is carried from input metadata only.

GCF_055597745.1	Lacticaseibacillus paracasei	No genome-wide ANI available; species label is carried from input metadata only.
GCF_055699145.1	Lacticaseibacillus paracasei	No genome-wide ANI available; species label is carried from input metadata only.
GCF_056619785.1	Lacticaseibacillus paracasei	No genome-wide ANI available; species label is carried from input metadata only.
GCF_056665955.1	Lacticaseibacillus paracasei	No genome-wide ANI available; species label is carried from input metadata only.
GCF_057418975.1	Lacticaseibacillus paracasei	No genome-wide ANI available; species label is carried from input metadata only.
GCF_057978265.1	Lacticaseibacillus paracasei	No genome-wide ANI available; species label is carried from input metadata only.
GCF_058070825.1	Lacticaseibacillus paracasei	No genome-wide ANI available; species label is carried from input metadata only.
GCF_058134255.1	Lacticaseibacillus paracasei	No genome-wide ANI available; species label is carried from input metadata only.

Table S3. Pangenome category counts and focal-isolate shared and strain-associated gene-content summary.

**Metric**	**Value**
Number of genomes	44
Core gene clusters (99-100%)	1848
Soft-core gene clusters (95-<99%)	230
Shell gene clusters (15-<95%)	1528
Cloud gene clusters (<15%)	3607
Total pangenome clusters	7213
Core + soft-core clusters	2078
Shell + cloud clusters	5135
Clusters shared by WU0904 and WU2302	2496
Clusters detected only in WU0904 relative to WU2302	380
Clusters detected only in WU2302 relative to WU0904	360
Shared WU clusters absent from all 42 public genomes	2

Table S4. Predicted processing architecture and similarity of focal WU0904/WU2302 bacteriocin candidates to reviewed bacteriocins.

**WU candidate(s)**	**Precursor length (aa)**	**Predicted leader (aa)**	**Predicted mature peptide (aa)**	**Closest reviewed mature bacteriocin**	**Global identity (%)**	**Interpretation**
PDMMEO_01282	66	20	46	O07623; subtilosin-A	28.26	Divergent predicted precursor; mature peptide remains weakly similar to reviewed bacteriocins.
ECDLDI_00178	66	20	46	Q48509; lactacin-F subunit LafX	31.25	Divergent predicted precursor; differs from PDMMEO_01282 at K22R, L60F, and V63D.
PDMMEO_01283 / ECDLDI_00177	84 / 84	18 / 18	66 / 66	Q48509; lactacin-F subunit LafX	28.79	Single precursor substitution (N8S) lies in the predicted leader; predicted mature peptides are identical.
PDMMEO_00357 / ECDLDI_01460	71 / 71	21 / 21	50 / 50	P24022; lactacin-F subunit LafA	31.58	Precursors and predicted mature peptides are identical between WU0904 and WU2302.
PDMMEO_00358 / ECDLDI_01459	77 / 101	19 / 43	58 / 58	P05834; microcin B17	29.31	WU2302 Bakta annotation contains a 24-aa N-terminal extension upstream of the same GG motif; predicted mature peptides are identical.
ECDLDI_01475 (group_2723)	59	20	39	P35618; sakacin-P	27.91	Divergent predicted class II-like accessory precursor; processing assignment is sequence-based and requires experimental validation.

Note: Seventy reviewed UniProt bacteriocin records (20-150 aa) were screened; 36 entries with explicit mature-peptide annotations were used for mature-peptide comparisons. The reported match is the reviewed mature bacteriocin with the highest conservative global identity for each WU candidate (ties omitted for brevity). Global identity was calculated as identical residues divided by the longer of the two mature-peptide sequences. WU cleavage sites were predicted from N-terminal double-glycine motifs and have not been experimentally validated. These comparisons support sequence divergence from reviewed mature bacteriocins and do not by themselves establish

Supplementary Table S5. Pairwise amino-acid comparison and revised interpretation of bacteriocin-associated proteins in WU0904 and WU2302.

WU0904 locus tag	WU2302 locus tag	Revised annotation	WU0904-WU2302 comparison	Interpretation / evidence status
PDMMEO_01282	ECDLDI_00178	Predicted bacteriocin precursor	95.455% identity over 66 aa	Three precursor substitutions: K22R, L60F, and V63D. Predicted leader length = 20 aa; predicted mature length = 46 aa.
PDMMEO_01283	ECDLDI_00177	Blp-family class II bacteriocin precursor	98.810% identity over 84 aa	Single N8S substitution lies within the predicted 18-aa leader; predicted 66-aa mature peptides are identical.
PDMMEO_00346	ECDLDI_01472	Bacteriocin-associated peptide	100% identity over 57 aa	Fully conserved between WU0904 and WU2302.
PDMMEO_00347	ECDLDI_01471	ComC/BlpC-family peptide	100% identity over 57 aa	Fully conserved between WU0904 and WU2302.
PDMMEO_00352	ECDLDI_01465	Bacteriocin immunity protein	98.182% identity over 110 aa	Conserved immunity-associated protein.
PDMMEO_00354	ECDLDI_01463	Bacteriocin immunity-like protein	98.198% identity over 111 aa	Uniform Bakta re-annotation called these proteins 'prebacteriocin'; however, the corresponding CBA7104

WU0904 locus tag	WU2302 locus tag	Revised annotation	WU0904-WU2302 comparison	Interpretation / evidence status
				RefSeq homolog WP_003576308.1 is annotated as a bacteriocin immunity protein. No canonical double-glycine motif was detected; excluded from precursor-processing analysis.
PDMMEO_00357	ECDLDI_01460	Class II bacteriocin precursor with double-glycine leader	100% identity over 71 aa	Predicted 21-aa leaders and 50-aa mature peptides; precursor and predicted mature sequences are identical.
PDMMEO_00358	ECDLDI_01459	Class II bacteriocin precursor with double-glycine leader	98.701% identity across the shared 77 aa; WU2302 Bakta prediction = 101 aa	WU2302 contains a 24-aa N-terminal extension upstream of the conserved GG motif. Alternative precursor starts imply leaders of 19 versus 43 aa but the same predicted 58-aa mature peptide. Translation initiation and cleavage require experimental validation.
NA	ECDLDI_01475	Divergent predicted class II-like accessory precursor (group_2723)	59 aa; identical orthologs also occur in DmW_181 and CBA7104	Predicted 20-aa N-terminal leader ending in GG and 39-aa mature peptide. Processing assignment is sequence-based and requires experimental validation.

Note: Product assignments were re-evaluated using uniform Bakta annotations, direct sequence inspection, the mature-peptide comparison, and the native RefSeq annotation of CBA7104 where relevant. Predicted cleavage sites and mature peptides are sequence-based and have not been experimentally verified. The 111-aa PDMMEO_00354/ECDLDI_01463 homolog was not treated as a bacteriocin precursor because it lacks a canonical double-glycine processing motif and the corresponding CBA7104 RefSeq homolog (WP_003576308.1) is annotated as a bacteriocin immunity protein.

Tables S6a. Genome-level bacteriocin-trait presence/absence matrix

Genome	Complete large-locus backbone	Small Blp-family locus	Rare accessory bacteriocin
GCF_051123965.1__M8	1	1	0
GCF_053749055.1__PL0013	1	0	0
GCF_014905075.1__NFFJ04	1	0	0
GCF_030540905.1__11w	0	0	0
GCF_039615415.1__TBM_27	1	0	0
GCF_013307125.1__Lp02	1	1	0
GCF_002902825.1__HDS-01	1	1	0
GCF_056665955.1__NIBL1583	1	0	0
GCF_012955485.1__347-16	1	1	0
GCF_028613785.1__H0104	0	1	0
GCF_003966835.1__IJH-SONE68	0	0	0
GCF_057978265.1__M501	1	0	0
GCF_049201625.1__UBLPc-87	1	0	0
GCF_052904645.1__F50	1	0	0
GCF_041117985.1__MYA5	1	0	0
GCF_001244395.1__L9	1	1	0
GCF_022701315.1__WX322	1	1	0
GCF_056619785.1__JM053	1	1	0
GCF_002079285.1__IIA	1	1	0
GCF_057418975.1__LK10	1	1	0
GCF_000829035.1__GTDB_reference	0	0	0
GCF_055597705.1__E-25	0	0	0
GCF_046581445.1__BBM396	1	1	0
GCF_040459685.1__T0601	1	1	0
GCF_052588795.2__S1	1	1	0
GCF_030418055.1__PWN_T9	1	1	0

WU0904_local	1	1	0
GCF_052601095.1__YWC-01	1	1	0
GCF_035330705.1__S135-24W	1	0	0
WU2302_local	1	1	1
GCF_002115655.1__DmW_181	1	1	1
GCF_048500655.1__CBA7104	1	0	1
GCF_000309605.1__32G	1	0	0
GCF_031461095.1__KCKM_0992	1	0	0
GCF_047785155.1__SRCM212209	1	0	0
GCF_044740695.1__E11.5	0	0	0
GCF_055699145.1__RF7	1	0	0
GCF_000376145.1__UW4	0	0	0
GCF_050320215.1__LTH_2407	0	0	0
GCF_049532865.1__PC-01	0	0	0
GCF_046116425.1__M115-8W-2	1	0	0
GCF_025190485.1__CIRM-BIA_1869	1	0	0
GCF_058070825.1__Lc19	1	0	0
GCF_051514555.1__ZFM847	0	0	0

Table S6b phylogenetic-clustering statistics.

Trait	Positive genomes	Observed mean patristic distance	Permutation mean distance	Distance clustering P	Observed Fitch changes	Permutation mean Fitch changes	Fitch clustering P
Complete large-locus backbone	34	0.281667	0.286192	0.151085	6	9.1656	0.005899
Small Blp-family locus	18	0.249989	0.28621	0.0024	7	14.1915	0.0002
Rare accessory bacteriocin	3	0.023641	0.286459	0.0002	1	2.9553	0.0004

Table S7. Software, commands, parameters, evidence status, and unresolved reproducibility items.

Analysis step	Software/version	Key parameters or evidence	Status
Public genome metadata retrieval	NCBI Datasets CLI; version not recovered	15 July 2026; --assembly-source all; --assembly-version current; --exclude-atypical; --mag exclude	Confirmed except version

Initial screening	Custom Python metadata parser	Genome size 2.5-3.6 Mb; contigs <=300; ordered niche-keyword matching; RefSeq preference	Confirmed
Species filter	skani-derived ANI/AF matrices; exact generation command not recovered	ANI >=95.0%; minimum directional AF >=50.0%; reference GCF_000829035.1	Thresholds confirmed
Dereplication	Custom filter_and_dereplicate.py	ANI >=99.9%; minimum directional AF >=50.0%; WU isolates forced retained	Confirmed
Final assembly QC	QUAST v5.3.0	--min-contig 500; genome size 2.5-3.6 Mb; contigs <=300; N50 >=10 kb; GC 45-48%	Confirmed
Local-genome taxonomy	GTDB-Tk v2.4.1; database r226	classify_wf; closest reference GCF_000829035.1	Confirmed
Uniform annotation	Bakta; version/database release to be inserted	Uniform annotation of 44 genomes	Version pending
Pangenome	Panaroo; version/options to be inserted	Core 99-100%; soft core 95-<99%; shell 15-<95%; cloud <15%	Version/options pending
Biosynthetic loci	antiSMASH 8.0	Region-level comparison; no significant known-cluster matches for focal RiPP-like loci	Confirmed

Synteny	clinker; version to be inserted	Curated locus comparison after orientation standardization	Version pending
Protein alignment	MAFFT v7.526	--auto	Confirmed
Phylogenetic trait analysis	Biopython/custom Python	10,000 permutations; random seed 42; mean patristic distance and Fitch parsimony	Confirmed
